# Honokiol relieves hippocampal neuronal damage in Alzheimer's disease by activating the SIRT3‐mediated mitochondrial autophagy

**DOI:** 10.1111/cns.14878

**Published:** 2024-08-04

**Authors:** Haitao Li, Jinmei Sun, Yili Wu, Yishu Yang, Wei Zhang, Yuanruhua Tian

**Affiliations:** ^1^ Department of Neurology, Beijing Friendship Hospital Capital Medical University Beijing China; ^2^ Zhejiang Provincial Clinical Research Center for Mental Disorders, School of Mental Health and The Affiliated Wenzhou Kangning Hospital, Institute of Aging, Key Laboratory of Alzheimer's Disease of Zhejiang Province Wenzhou Medical University, Oujiang Laboratory (Zhejiang Lab for Regenerative Medicine, Vision and Brain Health) Wenzhou China

**Keywords:** Alzheimer's disease, honokiol, mitochondrial autophagy, neuronal damage, SIRT3

## Abstract

**Background:**

This work elucidated the effect of honokiol (HKL) on hippocampal neuronal mitochondrial function in Alzheimer's disease (AD).

**Methods:**

APP/PS1 mice were used as AD mice models and exposed to HKL and 3‐TYP. Morris water maze experiment was performed to appraise cognitive performance of mice. Hippocampal Aβ+ plaque deposition and neuronal survival was evaluated by immunohistochemistry and Nissl staining. Hippocampal neurons were dissociated from C57BL/6 mouse embryos. Hippocampal neuronal AD model was constructed by Aβ oligomers induction and treated with HKL, CsA and 3‐TYP. Neuronal viability and apoptosis were detected by cell counting kit‐8 assay and TUNEL staining. mRFP–eGFP–LC3 assay, MitoSOX Red, dichlorodihydrofluorescein diacetate, and JC‐1 staining were performed to monitor neuronal autophagosomes, mitochondrial reactive oxygen species (ROS), neuronal ROS, and mitochondrial membrane potential. Autophagy‐related proteins were detected by Western blot.

**Results:**

In AD mice, HKL improved cognitive function, relieved hippocampal Aβ_1–42_ plaque deposition, promoted hippocampal neuron survival, and activated hippocampal SIRT3 expression and mitochondrial autophagy. These effects of HKL on AD mice were abolished by 3‐TYP treatment. In hippocampal neuronal AD model, HKL increased neuronal activity, attenuated neuronal apoptosis and Aβ aggregation, activated SIRT3 and mitochondrial autophagy, reduced mitochondrial and neuronal ROS, and elevated mitochondrial membrane potential. CsA treatment and 3‐TYP treatment abrogated the protection of HKL on hippocampal neuronal AD model. The promotion of mitochondrial autophagy by HKL in hippocampal neuronal AD model was counteracted by 3‐TYP.

**Conclusions:**

HKL activates SIRT3‐mediated mitochondrial autophagy to mitigate hippocampal neuronal damage in AD. HKL may be effective in treating AD.

## Graphical abstract

HKL improves cognitive performance in Alzheimer's disease mice by activating the SIRT3‐mediated mitochondrial autophagy.

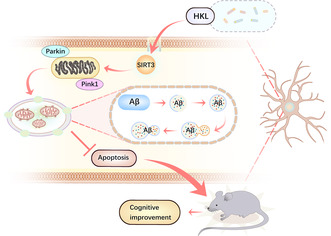

## 1 INTRODUCTION

Alzheimer's disease (AD), a common neurodegenerative disease, is characterized by progressive memory loss and cognitive and language impairment.^1^ In general, AD initiation and progression occur via amyloid‐β (Aβ) aggregation in neurons, and the removal of these neurotoxic Aβ aggregates has been proposed as an attractive strategy for treating AD.^2^, ^3^ AD usually develops in people over the age of 60 and is responsible for more than 90% of all dementia cases worldwide.^4^ AD negatively affects the quality of life of patients and causes an overload of physical and emotional stress on other family members and caregivers.^5^ In clinical settings, the main treatment strategy to relieve the symptoms associated with AD is the use of drugs; however, at present, the complete cure for AD is far from being achieved.^6^ Furthermore, effective therapy for AD is a grim challenge in the field of pharmaceutical sciences.

It has been identified that mitochondrial dysfunction is associated with the core pathological feature of AD as mitochondrial dysfunction leads to neuronal dysfunction and even massive neuronal death.^7^, ^8^ In AD, multiple mitochondrial abnormalities has been discovered, such as altered mitochondrial membrane potential, damaged mitochondrial structure, mitochondrial DNA (mtDNA) abnormalities, overproduction of mitochondrial reactive oxygen species (ROS), and reduced production of adenosine triphosphate (ATP).^9^ Besides, mitochondrial dysfunction in AD can disrupt the normal functioning of synapses, thereby resulting in the weakened synaptic connections and reduced neurotransmission; however, the weakened synaptic connections and reduced neurotransmission are important contributing factors to the main symptoms of AD, i.e., cognitive impairment and memory impairment.^10^ Treatment of AD has been a subject of intensive research in recent decades, and a focus on mitochondrial dysfunction would be helpful in developing effective therapeutic options for AD.

Honokiol (HKL) is a natural polyphenolic compound extracted from the Chinese herbal medicine *Magnolia officinalis*; it exhibits antioxidant and anti‐inflammatory properties.^11^ As a small molecule, HKL exerts a neuroprotective function by increasing its bioavailability in neurological tissues after crossing the blood–brain barrier.^12^, ^13^, ^14^ The potent antioxidant activity and anti‐neuroinflammatory properties of HKL can help alleviate neuronal excitotoxicity in the central nervous system.^14^ Furthermore, HKL displays an antidepressant effect, mitigating depression‐like symptoms in depressed mice by decreasing the secretion of proinflammatory factors and increasing the production of neuroprotective metabolites in hippocampal neurons.^15^ Moreover, it can alleviate neuronal damage and neurological deficits in rats after cerebral ischemia–reperfusion.^16^ In terms of neurodegenerative disease, HKL alleviated memory impairment, neuronal inflammation, and oxidative stress in rats with vascular dementia.^17^ In AD animals, HKL has been instructed to be effective in ameliorating cognitive impairment and alleviating Aβ deposition and neuroinflammation in the cortex and hippocampus.^18^ The ameliorative effect of HKL on memory impairment and its repression on glial cell activation have been suggested as primary ways to relieve AD.^19^ Besides, significantly suppressed Aβ plaque formation, neuroinflammation and neuronal loss have been discovered in AD rats treated with HKL.^20^ Thus, all of these previous findings suggest the neuroprotective function and therapeutic effect of HKL on AD. However, the exact molecular mechanisms by which HKL treats AD remain unelucidated to fully support its clinical application.

Importantly, recent studies have highlighted the ameliorative effect of HKL on mitochondrial dysfunction in AD. For instance, HKL has been suggested to block mitochondrial membrane potential loss in a dose‐dependent manner in AD mice, and also suppressed hippocampal neuronal apoptosis and reactive oxygen species generation.^21^ Further, in hippocampus of AD mice, HKL treatment distinctly suppressed the disturbed mitochondrial dynamics, mitochondrial dysfunction, and oxidative stress.^22^ Given the limited evidence of HKL alleviation on mitochondrial dysfunction in AD, this study was to delve deeper into the effects of HKL on mitochondrial dysfunction and the molecular mechanisms. This article will provide reliable basis for the clinical use of HKL in treating AD.

Sirtuin‐3 (SIRT3) is involved in the regulation of metabolism and oxidative stress; its deletion impairs neuronal activity and exacerbates the oxidative stress responses of neurons in AD.^23^ Interestingly, HKL can activate SIRT3.^24^ Recently, a study has reported that HKL can mitigate cognitive deficits in mice with AD by activating SIRT3 and improving mitochondrial function in the neurons.^25^ It is well known that mitochondrial dysfunction is a prominent driver involved in the progression of several neurodegenerative diseases, including AD.^26^ However, to date, data on whether HKL regulates AD progression by mediating neuronal mitochondrial dysfunction by activating SIRT3 remain scarce. Therefore, in the present study, we elucidated whether HKL regulates AD development by regulating SIRT3‐mediated mitochondrial dysfunction. Our study findings will provide a definitive basis for using HKL in the clinical treatment of AD.

## 2 MATERIALS AND METHODS

### 2.1 Animals and treatment

Male APP/PS1 mice (9 months old, *n* = 36) that purchased from Gene&Peacebiotech Co., Ltd. (Jiangsu, China) were used as animal AD models. Male wild‐type C57BL/6J mice (9 months old, *n* = 9) (Gene&Peacebiotech Co., Ltd., Jiangsu, China) were used as control, and set as the WT group. All mice were housed in a room free of specific pathogens at 22°C with a 12‐h day/night cycle. All mice had free access to food and water. The conduction of animal experiments was implemented with the permission of the Animal Ethics Committee (Approval Number: MDKN‐2023‐043). In this study, HKL was commercially supplied by Yuanye Biotechnology (Shanghai, China).

The 36 male APP/PS1 mice were randomly divided into four groups: the APP/PS1 group, the HKL‐L group, the HKL‐H group, and the HKL‐H+3‐TYP group, with nine mice per group. The nine male wild‐type C57BL/6J mice were used as the WT group. The treatment of mice in each group was as follows: mice of the HKL‐L group and the HKL‐H group were given HKL treatment by gavage at doses of 10 and 20 mg/kg/day for five consecutive days^25^, ^27^; for mice of the HKL‐H+3‐TYP group, they were intraperitoneally injected with 3‐TYP (50 mg/kg/day)^28^, ^29^ (an inhibitor of SIRT3) (BioLab Technology, Beijing, China) and administered with HKL (20 mg/kg/day) by gavage for 5 consecutive days; mice of the WT group and the APP/PS1 group were given an equal volume of normal saline (0.9% NaCl) by gavage every day. After 5 days of HKL treatment, 5% isoflurane (Reward Life Technology, Shenzhen, China) was adopted to induce deep anesthesia in mice. After euthanized, the whole brains of all mice were obtained and kept at −80°C.

### 2.2 Morris water maze experiment

In Morris water maze experiment, mice were placed in a tank divided equally into four quadrants. Among the four quadrants, one quadrant (i.e., the target quadrant) was selected and placed a platform 1 cm under the water surface. On days 1, 2, 3, 4 and 5 of HKL treatment, mice were placed on the opposite quadrant to the target quadrant. Within 60 s, the escape latency, number of platform crossing and latency to cross platform location of each mouse was recorded by a camera system connected to a computer. Mice were guided to find the platform if they did not reach the platform within 60 s. The water temperature was kept at 22 ± 1°C. It should be noticed that, before the formal trials, mice were trained to discover the platform for 3 days. The formal trials were implemented 24 h after the last training.^30^

### 2.3 Novel object recognition test

This study used the novel objective recognition test to appraise the recognition memory of mice. The test included two phases: familiarization phase and test phase. A 30‐min time interval was set between the two phases. In familiarization phase, mice were placed in a box (40 cm in length, 24 cm in width, and 32 cm in height) to explore the cage and objects freely for 5 min. Two same objects (same color and shape) were placed 10 cm from the edge of the box. In test phase, mice were placed back to the box in which one object was replaced by a novel object. The novel object was different in color and shape from the original one, but similar in size to it. Mice moved freely in the box for 5 min. The trajectory of mice was recorded by a video camera. The time that the nose‐points of mice directed towards the objects 3 cm away was considered as the exploration time. Recognition memory was defined as discrimination index that calculated by (time to explore the novel object‐time to explore the original object)/(time to explore the novel object + time to explore the original object).^31^

### 2.4 Congo‐red staining and Nissl staining

The whole brains of mice were fixed in 4% paraformaldehyde (Solarbio Technology, Beijing, China). After paraffin‐embedded, the whole brains were prepared into slices (5 μm). The slices were dewaxed by xylene, followed by being rehydrated with gradient alcohol. Congo‐red staining solution (Solarbio Technology, Beijing, China) was used to staining the slices for 15 min. The remaining staining solution was washed away by distilled water. After differentiated by alkaline alcohol solution, the slices underwent counterstaining with hematoxylin staining solution (Solarbio Technology, Beijing, China). Following dehydration, neutral resin was used to enclose the slices. The observation and image acquisition of plaque deposition in hippocampus and cortex was observed under an optical microscope (Olympus, Tokyo, Japan).^32^

Regarding Nissl staining, the rehydrated sections were stained with Nissl staining solution (Yansheng Industry, Shanghai, China) for 40 min at 60°C. Subsequently, 0.5% acid alcohol differentiation solution (Yansheng Industry, Shanghai, China) was adopted to react with the slices for 2 min. After dehydrated, the slices were placed under an optical microscope (Olympus, Tokyo, Japan) for image acquisition. Besides, the number of Nissl bodies in hippocampus was counted.^33^

### 2.5 Immunohistochemistry

After dewaxed and rehydrated, the whole brain slices underwent antigen repair in boiled sodium citrate buffer for 10 min, and then blockage in 5% normal goat serum (Xuanya Biotechnology, Shanghai, China) for 30 min at room temperature. Rabbit anti‐Aβ_1–42_ primary antibody (1:100, kl957Ra01, Kanglang Biotechnology, Shanghai, China) was adopted to react with the slices for 12 h at 4°C. Then goat anti‐rabbit secondary antibody that had been labeled with horseradish peroxidase (1:200, ab97051, Abcam, Shanghai, China) was dropped to the slices for 30 min reaction at room temperature. Followed by this, 3,3′‐diaminobenzidine (Xinfan Biotechnology, Shanghai, China) added onto the slices for 5 min color reaction. The slices were dehydrated and enclosed, and the number of Aβ_1–42_ plaques in hippocampus and cortex was counted under an optical microscope (Olympus, Tokyo, Japan).^34^

### 2.6 Immunofluorescence staining

The expression of LC3 protein in hippocampus and cortex of mice was examined by immunofluorescence staining. Neuronal nuclear antigen (NeuN) immunofluorescence staining was used for localization of neurons. In short, the whole brain slices that had been dewaxed and rehydrated were blocked in 10% bovine serum albumin for 30 min. Then rabbit anti‐LC3 primary antibody (1:100, abs155467, Absin Biotechnology, Shanghai, China), as well as mouse anti‐NeuN primary antibody (1:100, IPD‐ANM4324, Aipti Biological Engineering, Hubei, Wuhan), were dropped onto the slices for 12 h treatment at 4°C. In the next step, Alexa Fluor594‐conjugated goat anti‐rabbit secondary antibody (1:200, bs‐0295G‐AF594, Xinyu Biotechnology, Shanghai, China), as well as Alexa Fluor488‐conjugated goat anti‐mouse secondary antibody (1:200, 115‐545‐044, AmyJet Scientific, Wuhan, China) were adopted to probe the slices for 2 h at room temperature. After nuclear staining by 4′, 6‐diamidino‐2‐phenylindole (DAPI) (Biolab Technology, Beijing, China) treatment, the slices were placed under a fluorescence microscope (Olympus, Tokyo, Japan) foe the observation of LC3 and NeuN immunofluorescence staining.^35^

### 2.7 Isolation of primary mouse hippocampal neurons

Pregnant C57BL/6 mice were commercially supplied by Junke Bioengineering (Nanjing, China). All animal experiments were approved by the Animal Ethics Committee and followed the “Guiding Opinions on the Good Treatment of Laboratory Animals” issued by the Ministry of Science and Technology of the People's Republic of China.

On the 17th day of pregnancy, pregnant C57BL/6 mice were anesthetized using 5% isoflurane (Yuyan Scientific Instruments, Shanghai, China) and sacrificed via cervical dislocation after no response to head and limb stimulation. Mouse embryos were dissected from the maternal mice, followed by the collection of the hippocampal tissues of these mouse embryos. The hippocampal tissues were cut using a sterile scissor and then dissociated in 0.25% trypsin solution (Solarbio Technology, Beijing China) for 15 min at 37°C. The dissociated cells (5 × 10^4^ cells/well) were grown in six‐well plates coated with poly‐ʟ‐lysine and cultured in Neurobasal medium (Yubo Biotechnology, Shanghai, China) supplemented with l‐glutamine (2 mM, Solarbio Technology, Beijing, China), B27 supplement (Solarbio Technology, Beijing, China), and 1% penicillin/streptomycin (Solarbio Technology, Beijing, China). The Neurobasal medium was refreshed at intervals of 2 days. After culturing for 10 days at 37°C, under 5% CO_2_, these cells were used for subsequent experiments.^36^

### 2.8 Identification of primary mouse hippocampal neurons

To identify the isolated cells, we detected the expression of neuronal‐specific markers, including neuronal nuclei (NeuN) and microtubule‐associated protein 2 (MAP2), via immunofluorescence staining. The isolated cells were fixed with 4% paraformaldehyde for 15 min at room temperature. Then, they were blocked with 5% bovine serum albumin (Solarbio Technology, Beijing, China) for 30 min at room temperature. The cells were treated with rabbit anti‐NeuN (1:200, ab104225, Abcam, Shanghai, China) and mouse anti‐MAP2 (1:200, ab11267, Abcam, Shanghai, China) primary antibodies for 12 h at 4°C. Thereafter, cells were incubated with secondary antibodies for 2 h at room temperature; the secondary antibodies were Alexa Fluor488‐conjugated goat anti‐rabbit secondary antibody (1:300, A0423, Beyotime, Shanghai, China) and Alexa Fluor594‐conjugated goat anti‐mouse secondary antibody (1:300, D110102, Sangon Biotech, Shanghai, China). After staining the nucleus with DAPI (Biolab Technology, Beijing, China), the cells were observed under a fluorescence microscope (BX51, Olympus, Tokyo, Japan) for NeuN and MAP2 expression. Positive fluorescence staining of NeuN and MAP2 indicated the successful isolation of primary mouse hippocampal neurons.^37^

### 2.9 Preparation of AβOs

Aβ_1–42_ lyophilized powder was obtained from Lianmai Bioengineering (Shanghai, China). A homogenous suspension of Aβ_1–42_ lyophilized powder with a concentration of 1 mM was prepared by mixing it thoroughly with 1,1,1,3,3,3‐hexa‐fluoro‐2‐propanol (Haohong Biomedical Technology, Shanghai, China). Then, dimethyl sulfoxide (Solarbio Technology, Beijing, China) was added into the Aβ_1–42_ homogenous suspension (to a concentration of 5 mM). The mixture was subjected to ultrasonication for 10 min. Thereafter, the mixture was diluted to a concentration of 100 μM using phosphate‐buffered saline (PBS) (Solarbio Technology, Beijing, China) containing 0.05% sodium dodecyl sulfate (Beyotime, Shanghai, China). After incubation for 24 h at 4°C, PBS was readded to obtain a final concentration of 10 μM. The solution was incubated for 2 weeks at 4°C. AβOs were obtained by centrifugation for 10 min at 13,000 rpm and 4°C.^25^ To identify AβOs, Western blot was performed.

### 2.10 Treatment of primary mouse hippocampal neurons with AβOs, HKL, cyclosporine A (CsA), and 3‐(1H‐1,2,3‐triazol‐4‐yl) pyridine (3‐TYP)

Primary mouse hippocampal neurons were plated into six‐well plates at a density of 1 × 10^5^ hippocampal neurons per well. AβOs at a concentration of 1 μM^25^ (diluted using Neurobasal medium) were used to treat primary mouse hippocampal neurons to construct the hippocampal neuronal model of AD. Furthermore, HKL at concentrations of 5 and 10 μM (diluted using Neurobasal medium) was used to treat normal and AβO‐induced primary mouse hippocampal neurons, respectively. For treatment with CsA (a mitochondrial autophagy inhibitor) and 3‐TYP (SIRT3 inhibitor), 1 μM^38^ CsA (diluted using Neurobasal medium) (Shanran Biotechnology, Shanghai, China) and 50 μM^39^ 3‐TYP (diluted using Neurobasal medium) (Biolab Technology, Beijing, China) were added to pretreated primary mouse hippocampal neurons for 1 h at 37°C, under 5% CO_2_. Thereafter, AβO induction and HKL treatment were performed. Primary mouse hippocampal neurons cultured under normal conditions were used as the control.

### 2.11 Cell Counting Kit‐8 (CCK‐8) assay

Primary mouse hippocampal neurons were inoculated into 96‐well plates at a density of 5 × 10^3^ hippocampal neurons per 100 μL of the relevant medium. After culturing for 24 and 48 h at 37°C and 5% CO_2_, the hippocampal neurons in each group were treated with 10 μL of the CCK‐8 solution (Solarbio Technology, Beijing, China) for 2 h at 37°C. The absorbance was measured at a wavelength of 490 nm using an ultramicroplate reader (EL808, BioTek, Winooski, VT, USA). The cell viability was determined by referring to the absorbance of the control.^40^

### 2.12 Determination of lactate dehydrogenase (LDH) activity, and content of malondialdehyde (MDA), superoxide dismutase (SOD), and ROS

After 48 h of treatment under the relevant conditions, the culture medium in the primary mouse hippocampal neurons of each group was collected and centrifuged for 10 min at 10,000 rpm and 4°C. The LDH activity in the supernatant was determined using an LDH activity assay kit (Solarbio Technology, Beijing, China) according to the manufacturer's instructions. Further, the levels of MDA, SOD and ROS in hippocampus of mice were detected by according to MDA assay kit (Beyotime, Shanghai, China), SOD assay kit (Beyotime, Shanghai, China), and ROS assay kit (Beyotime, Shanghai, China).^41^

### 2.13 Terminal deoxynucleotidyl transferase dUTP nick end labeling (TUNEL) staining

The primary mouse hippocampal neurons in each group were grown for 48 h in the relevant medium, followed by being fixed with 4% paraformaldehyde for 15 min at room temperature. TUNEL staining was performed by using the One‐Step TUNEL Apoptosis Assay Kit (Beyotime, Shanghai, China) according to the manufacturer's instructions. After permeabilization with 0.1% Triton X‐100 (Beyotime, Shanghai, China) for 10 min at room temperature, hippocampal neurons were treated with the TUNEL working solution (Beyotime, Shanghai, China) for 1 h at 37°C and then with DAPI solution for 5 min at room temperature. Finally, hippocampal neurons were placed under a fluorescence microscope (BX51, Olympus, Tokyo, Japan) for detection of apoptosis.^37^

### 2.14 Detection of Aβ_1–42_ concentration

The primary mouse hippocampal neurons in each group were harvested after culturing for 48 h and lysed using a lysate solution (Solarbio Technology, Beijing, China) for 30 min on ice. The supernatant was obtained by centrifuging the samples for 10 min at 4°C. Aβ_1–42_ concentration in the supernatant was measured by using an Aβ_1–42_ assay kit (Xinyu Biotechnology, Shanghai, China) according to the manufacturer's instructions.^42^

### 2.15 mRFP–eGFP–LC3 assay

After culturing for 48 h under the relevant conditions, the primary mouse hippocampal neurons in each group were subjected to transfection with the mRFP–EGFP–LC3 plasmid (Xingtuo Biomedical Technology, Shanghai, China) according to the manufacturer's instructions. After transfection, hippocampal neurons were cultured for 24 h at 37°C and 5% CO_2_, followed by being fixed with 4% paraformaldehyde for 15 min and staining with DAPI for 5 min at room temperature. The number of autophagosomes was determined under a fluorescence microscope (BX51, Olympus) by counting the number of LC3 puncta.^43^

### 2.16 Transmission electron microscopy (TEM)

The primary mouse hippocampal neurons in each group were cultured for 48 h and then fixed with 4% paraformaldehyde for 15 min at room temperature. After treatment with 1% osmium tetroxide, the hippocampal neurons were dehydrated using an alcohol gradient and embedded into paraffin. Thereafter, hippocampal neurons were disclosed into neutral resin, followed by cutting them into sections with a thickness of 65 nm. The sections were stained with 2% uranyl acetate and lead citrate for 10 min at room temperature. Autophagosomes were observed under a TEM (H‐7000, Hitachi, Tokyo, Japan).^44^

### 2.17 MitoSOX Red staining and dichlorodihydrofluorescein diacetate (DCFH‐DA) staining

The primary mouse hippocampal neurons in each group were subjected to MitoSOX Red and DCFH‐DA staining to determine mitochondrial and neuronal ROS levels, respectively. The experiment procedure was performed as previously reported.^45^ Briefly, the hippocampal neurons in each group were cultured for 48 h under the relevant conditions and then stained with DCFH‐DA solution (Biolab Technology, Beijing, China) and MitoSOX Red solution (Lianqiao Biotechnology, Shanghai, China) for 30 min at 37°C. DAPI was used to stain the nucleus after MitoSOX Red staining. Mitochondrial and neuronal ROS levels were measured by evaluating the fluorescence intensity of DCFH‐DA and MitoSOX Red, as detected under the fluorescence microscope (BX51, Olympus, Tokyo, Japan). Fluorescence intensity was quantified using Image‐Pro Plus software (Media Cybernetics, Bethesda, MD, USA).

### 2.18 JC‐1 staining combined with flow cytometry

JC‐1 staining combined with flow cytometry was performed to measure the mitochondrial membrane potential of primary mouse hippocampal neurons. After treatment for 48 h under the relevant conditions, the hippocampal neurons in each group were harvested and stained with JC‐1 solution (Biolab Technology, Beijing, China) for 20 min at 37°C. Thereafter, the FACSort flow cytometer (Becton Dickinson, San Jose, CA, USA) was used to detect JC‐1 polymers and JC‐1 monomers. Data were analyzed using FlowJo software (Tree Star Inc., Ashland, OR, USA). JC‐1 can aggregate to form polymers in healthy mitochondria; however, it is present as monomers in damaged mitochondria. Therefore, the ratio of JC‐1 polymers/monomers can reflect the changes in mitochondrial membrane potential.^45^

### 2.19 Determination of ATP content

The primary mouse hippocampal neurons in each group were cultured for 48 h under the relevant conditions and then lysed with lysis buffer (Beyotime, Shanghai, China) for 30 min on ice. Additionally, the hippocampus was also lysed with lysis buffer. After centrifuging the lysis mixture samples for 10 min at 12,000 rpm and 4°C, the supernatant was collected for ATP measurement using an ATP assay kit (Biolab Technology, Beijing, China). The detection procedure was performed according to the manufacturer's instructions.^42^

### 2.20 Real‐time quantitative reverse transcription polymerase chain reaction (qRT‐PCR)

To measure mtDNA expression, mtDNA was extracted from the primary mouse hippocampal neurons and hippocampus in each group, which were cultured for 48 h under the relevant conditions, using the Mitochondrial DNA Isolation Kit (AmyJet Scientific, Wuhan, China). The PrimeScript RT Reagent Kit (Takara, Ohtsu, Shiga, Japan) was used to synthesize complementary DNA according to the manufacturer's directions. PCR was performed using the SYBR Premix Ex Taq II (TaKaRa, Shiga, Japan) on the 7500 Real‐Time PCR System (Applied Biosystems, Foster City, CA, USA). The PCR conditions were as follows: 40 cycles at 95°C for 30 s, 50°C for 30 s, and 72°C for 30 s. The relative mRNA expression of mtDNA was evaluated using the $2^{-∆∆C_{T}}$ method, with mitochondrial D‐loop as the target gene and 18S rRNA as the control. Primers were designed and synthesized by GeneChem (Shanghai, China). The primer sequences were as follows: D‐loop, forward: 5′‐AAGTGGCTGTGCAGACATTC‐3′ and reverse: 5′‐TCTGTCTTTGATTCCTGCCT‐3′, and 18S rRNA, forward: 5′‐TCTCCTACTTGGATAACTGTGG‐3′ and reverse: 5′‐GGCGACTACCATCGAAAGTTG‐3′.^46^

### 2.21 Translocase of outer mitochondrial membrane 20 (TOMM20)/Parkin immunofluorescence staining

TOMM20/Parkin immunofluorescence staining was performed to determine the expression of Parkin in the mitochondria. Briefly, primary mouse hippocampal neurons were cultured for 48 h under the relevant conditions and fixed with 4% paraformaldehyde for 10 min at room temperature. Then, hippocampal neurons were permeabilized with 0.1% Triton X‐100 (Solarbio Technology, Beijing, China) for 10 min and blocked with normal goat serum (Solarbio Technology, Beijing, China) for 30 min at room temperature. Thereafter, rabbit anti‐TOMM20 (1:100, ab78547, Abcam, Shanghai, China) and mouse anti‐Parkin (1:100, ab77924, Abcam, Shanghai, China) were used to probe these hippocampal neurons overnight at 4°C. Alexa Fluor594‐conjugated goat anti‐rabbit (1:200, 111–585–003, AmyJet Scientific, Wuhan, China) and Alexa Fluor488‐conjugated goat anti‐mouse (1:200, 115–545–044, AmyJet Scientific, Wuhan, China) secondary antibodies were added for a 2‐h reaction at room temperature. The fluorescence intensities of TOMM20 and Parkin were observed under a fluorescence microscope (BX51, Olympus, Tokyo, Japan).^47^

### 2.22 Western blot

After culturing for 48 h under the relevant conditions, the primary mouse hippocampal neurons in each group were harvested to extract total protein using radioimmunoprecipitation assay (RIPA) lysis buffer (Solarbio Technology, Beijing, China). The Cellular Mitochondrial Isolation Kit (Beyotime, Shanghai, China) was used to extract the mitochondria in the hippocampal neurons in each group. Besides, total proteins in mice hippocampus was also extract by RIPA lysis buffer. Animal Tissue Mitochondrial Extraction Kit (Biolab Technology, Beijing, China) was adopted for mitochondria extraction in mice hippocampus. Mitochondrial proteins were extracted using RIPA lysis buffer. After separation using 10% sodium dodecyl sulfate–polyacrylamide gel electrophoresis, the proteins were transferred onto polyvinylidene fluoride (PVDF) membranes for blocking with 5% skimmed milk (Solarbio Technology, Beijing, China) for 1 h at room temperature. Subsequently, primary antibodies were used to probe these proteins for 12 h overnight. The primary antibodies were rabbit anti‐SIRT3 (ab217319, Abcam, Shanghai, China), rabbit anti‐GAPDH (1:1000, ab9485, Abcam, Shanghai, China), rabbit anti‐LC3 (1:1000, abs155467, Absin Biotechnology, Shanghai, China), rabbit anti‐Parkin (1:1000, ab73016, Abcam, Shanghai, China), rabbit anti‐PINK1 (1:1000, ab300623, Abcam, Shanghai, China), rabbit anti‐P62 (1:1000, 16‐RB62, Yubo Biotechnology, Shanghai, China), and rabbit anti‐COXIV (1:1000, ab202554, Abcam, Shanghai, China). To identify the prepared AβO samples, rabbit anti‐Aβ primary antibody (ABP57498, AmyJet Scientific, Wuhan, China) was used. Next, the PVDF membranes were incubated with goat anti‐rabbit secondary antibody (1:2000, ab97051, Abcam, Shanghai, China) for 2 h at room temperature. Protein blots were visualized after treatment with an enhanced chemiluminescence reagent (Solarbio Technology, Beijing, China). Finally, Image‐Pro Plus software (Media Cybernetics) was used to quantify these protein blots. The relative expression of the proteins in the hippocampal neurons was normalized to that of GAPDH, whereas the relative expression of mitochondrial proteins was calculated by referring to COXIV.^48^

### 2.23 Statistical analysis

Statistical analysis of the data (represented as mean ± standard deviation) was performed using GraphPad Prism 6.0 statistical software (GraphPad Software Inc., San Diego, CA, USA). The Kolmogorov–Smirnov and the Shapiro–Wilk tests were utilized for normal distribution of data. All data in the current work showed a normal distribution. One‐way analysis of variance combined with post‐hoc Tukey's test was used to compare differences in more than two groups. A *p*‐value of <0.05 indicates a statistically significant difference.

## 3 RESULTS

### 3.1 HKL improved cognitive performance of AD mice, reduced Aβ_1–42_ plaque deposition in hippocampus and cortex, and enhanced hippocampal neuronal survival in AD mice

**FIGURE 1:**
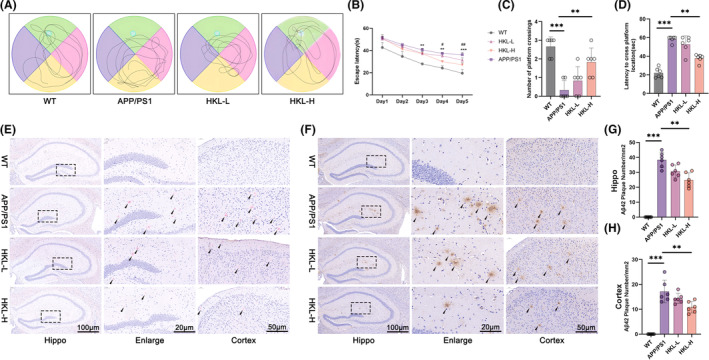
HKL was effective in improving cognitive performance of AD mice, and reducing Aβ_1–42_ plaque deposition in hippocampus and cortex in AD mice. (A–D) Morris water maze experiment supported the effectiveness of HKL in improving cognitive performance of AD mice. **p < 0.01, and ***p < 0.001 versus the WT group. ^#^ *p* < 0.05, and ^##^ *p* < 0.01 versus the HKL‐H group. (E) Congo‐red staining implied the suppression of HKL on plaque deposition in hippocampus and cortex of AD mice. (F–H) Immunohistochemistry revealed the blockage of HKL on Aβ_1–42_ plaque deposition in AD mice. **p < 0.01, ***p < 0.001.

This work adopted APP/PS1 mice as animal models of AD and treated them with low‐ and high‐ doses of HKL. Wild‐type C57BL/6J mice were served as control (named the WT group). The influence of HKL on cognitive performance in APP/PS1 mice was appraised by morris water maze experiment. APP/PS1 mice displayed significantly prolonged escape latency and latency to cross platform location, as well as lower number of platform crossing, as relative to wild‐type C57BL/6J mice (*p* < 0.01 or *p* < 0.001). In fact, high dose of HKL treatment prominently improved cognitive performance in APP/PS1 mice, as supported by the shortened escape latency and latency to cross platform location, and the higher number of platform crossing (the HKL‐H group vs. the APP/PS1 group) (*p* < 0.05 or *p* < 0.01) (Figure 1A–D) (Figure 1B: *F* = 25.59, df = 3; Figure 1C: *F* = 15.60, df = 3; Figure 1D: *F* = 45.26, df = 3).

To examine plaque deposition, Congo‐red staining was implemented on the hippocampus and cortex of mice. Plaque deposition was not observed in hippocampus and cortex of wild‐type C57BL/6J mice. However, APP/PS1 mice showed severe plaque deposition in their hippocampus and cortex. Interestingly, both low and high doses of HKL treatments, especially high dose of HKL treatment, were effective in reducing plaque deposition in hippocampus and cortex of APP/PS1 mice (Figure 1E). Additionally, Aβ_1–42_ plaques in hippocampus and cortex of mice were determined by immunohistochemistry. Consequently, a large number of Aβ_1–42_ plaques were discovered in APP/PS1 mice, as matched to wild‐type C57BL/6J mice (*p* < 0.001). Conversely, high dose of HKL treatment pronouncedly reduced number of Aβ_1–42_ plaques in hippocampus and cortex of APP/PS1 mice (the HKL‐H group vs. the APP/PS1 group) (*p* < 0.01) (Figure 1F–H) (Figure 1G: *F* = 44.89, df = 3; Figure 1H: *F* = 97.25, df = 3). Thus, HKL was effective in improving cognitive performance, and reducing Aβ_1–42_ plaque deposition in hippocampus and cortex of AD mice.

Discrimination index that acquired from the novel object recognition test was used to assess the recognition memory of mice. A decrease in discrimination index was found in APP/PS1 mice (the APP/PS1 group vs. the WT group) (*p* < 0.01), whereas high dose of HKL treatment significantly increased discrimination index of APP/PS1 mice (the HKL‐H group vs. the APP/PS1 group) (*p* < 0.05) (Figure S1A,B) (Figure S1B: *F* = 51.85, df = 3). Nissl staining revealed a much reduction in Nissl body number in APP/PS1 mice hippocampal CA1 region, than wild‐type C57BL/6J mice (*p* < 0.001). Conversely, HKL treatments (the HKL‐L group and the HKL‐H group) led to an elevation in Nissl body number in hippocampal CA1 region of APP/PS1 mice, as referring to the APP/PS1 group (*p* < 0.05 or *p* < 0.01) (Figure S1C,D) (Figure S1D: *F* = 126.7, df = 3). Therefore, HKL treatment improved recognition memory of AD mice, and facilitated neuronal survival in hippocampus of AD mice.

### 3.2 HKL activated hippocampal SIRT3 expression and hippocampal mitochondrial autophagy in AD mice

**FIGURE 2:**
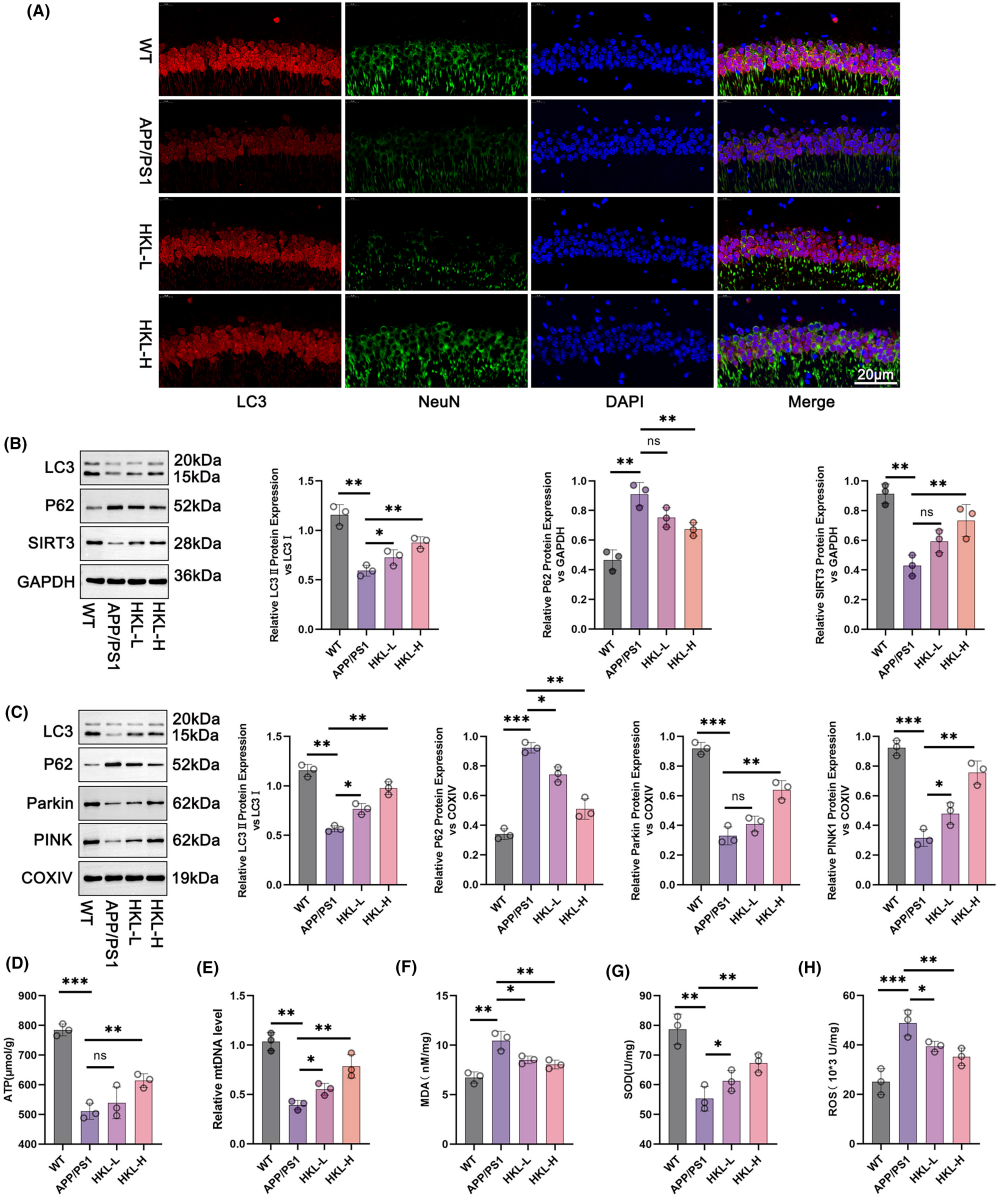
HKL activated hippocampal SIRT3 expression and hippocampal mitochondrial autophagy in AD mice. (A) Immunofluorescence staining indicated the promotion of HKL on hippocampal autophagy in AD mice. (B) The enhancement of HKL on hippocampal autophagy in AD mice was demonstrated by Western blot. (C) Western blot revealed the promotion of HKL on hippocampal mitochondrial autophagy in AD mice. (D–H) The protective effect of HKL on hippocampus and hippocampal mitochondria in AD mice was illustrated, as evidenced by its promotion on ATP, mtDNA, and SOD, as well as suppression on MDA and ROS. *p < 0.05, **p < 0.01, ***p < 0.001. “ns” represented differences that were not statistically significant.

To appraise the influence of HKL on neuronal autophagy, the expression of LC3 in hippocampus and cortex of mice was visualized by immunofluorescence staining. Diminished intensity of LC3 staining occurred in hippocampus and cortex of APP/PS1 mice, in contrast to wild‐type C57BL/6J mice. In fact, APP/PS1 mice of the two HKL treatment groups exhibited a much enhancement in LC3 staining intensity, comparatively (the HKL‐L group vs. the APP/PS1 group; the HKL‐H group vs. the APP/PS1 group) (Figure 2A, Figure S1E). This phenomenon was indicative that HKL was effective in activating neuronal autophagy in AD mice.

Hippocampal damage is a major contributor to cognitive dysfunction in AD. Thus, in the subsequent study, the effect of HKL on hippocampal neuronal autophagy was scrutinized by Western blot. In comparison to wild‐type C57BL/6J mice, APP/PS1 mice expressed less LC3II/LC3I and SIRT3 proteins, but more P62 protein in hippocampus (*p* < 0.01). Comparatively, low dose of HKL treatment compensated LC3II/LC3I protein expression in hippocampus of APP/PS1 mice (the HKL‐L group vs. the APP/PS1 group) (*p* < 0.05). At the same time, both low and high doses of HKL treatments facilitated the expression of LC3II/LC3I and SIRT3 proteins, and blocked the expression of P62 protein in hippocampus of APP/PS1 mice (the HKL‐L group vs. the APP/PS1 group; the HKL‐H group vs. the APP/PS1 group) (*p* < 0.01) (Figure 2B: *F* = 30.05, df = 3 for LC3II/LC3I; *F* = 23.74, df = 3 for P62; *F* = 18.91, df = 3 for SIRT3). Further, to examine mitochondrial autophagy, mitochondria from hippocampal tissues were extracted, and the expression of mitochondrial autophagy‐related proteins was evaluated by Western blot. As a result, a reduction in the expression of LC3II/LC3I, Parkin and PINK1 proteins, combined with an increase in P62 protein expression, was discovered in APP/PS1 mice, as referring to wild‐type C57BL/6J mice (*p* < 0.01 or *p* < 0.001). HKL treatment led to an elevation in the expression of LC3II/LC3I, Parkin and PINK1 proteins and a decrease in the expression of P62 protein in hippocampal neuronal mitochondria of APP/PS1 mice, comparatively, (the HKL‐L group vs. the APP/PS1 group; the HKL‐H group vs. the APP/PS1 group) (*p* < 0.05 or *p* < 0.01) (Figure 2C: *F* = 65.77, df = 3 for LC3II/LC3I; *F* = 79.22, df = 3 for P62; *F* = 70.17, df = 3 for Parkin; *F* = 52.31, df = 3 for PINK1). The levels of ATP, MDA, SOD, and ROS in hippocampus of mice, as well as mtDNA in hippocampal neuronal mitochondria were tested, respectively. APP/PS1 mice showed lower ATP, mtDNA, and SOD levels, but higher MDA and ROS levels than wild‐type C57BL/6J mice (*p* < 0.01 or *p* < 0.001). Indeed, HKL treatment resulted in an elevation in ATP, mtDNA, and SOD levels, as well as a reduction in MDA and ROS levels in APP/PS1 mice (the HKL‐L group vs. the APP/PS1 group; the HKL‐H group vs. the APP/PS1 group) (*p* < 0.05 or *p* < 0.01) (Figure 2D–H) (Figure 2D: *F* = 39.87, df = 3; Figure 2E: *F* = 33.34, df = 3; Figure 2F: *F* = 18.22, df = 3; Figure 2G: *F* = 18.20, df = 3; Figure 2H: *F* = 16.14, df = 3). All of these data implied that HKL enhanced hippocampal SIRT3 expression, and activated hippocampal mitochondrial autophagy in AD mice.

### 3.3 HKL relieved hippocampal neuronal damage and intracellular Aβ aggregation in the hippocampal neuronal model of AD

**FIGURE 3:**
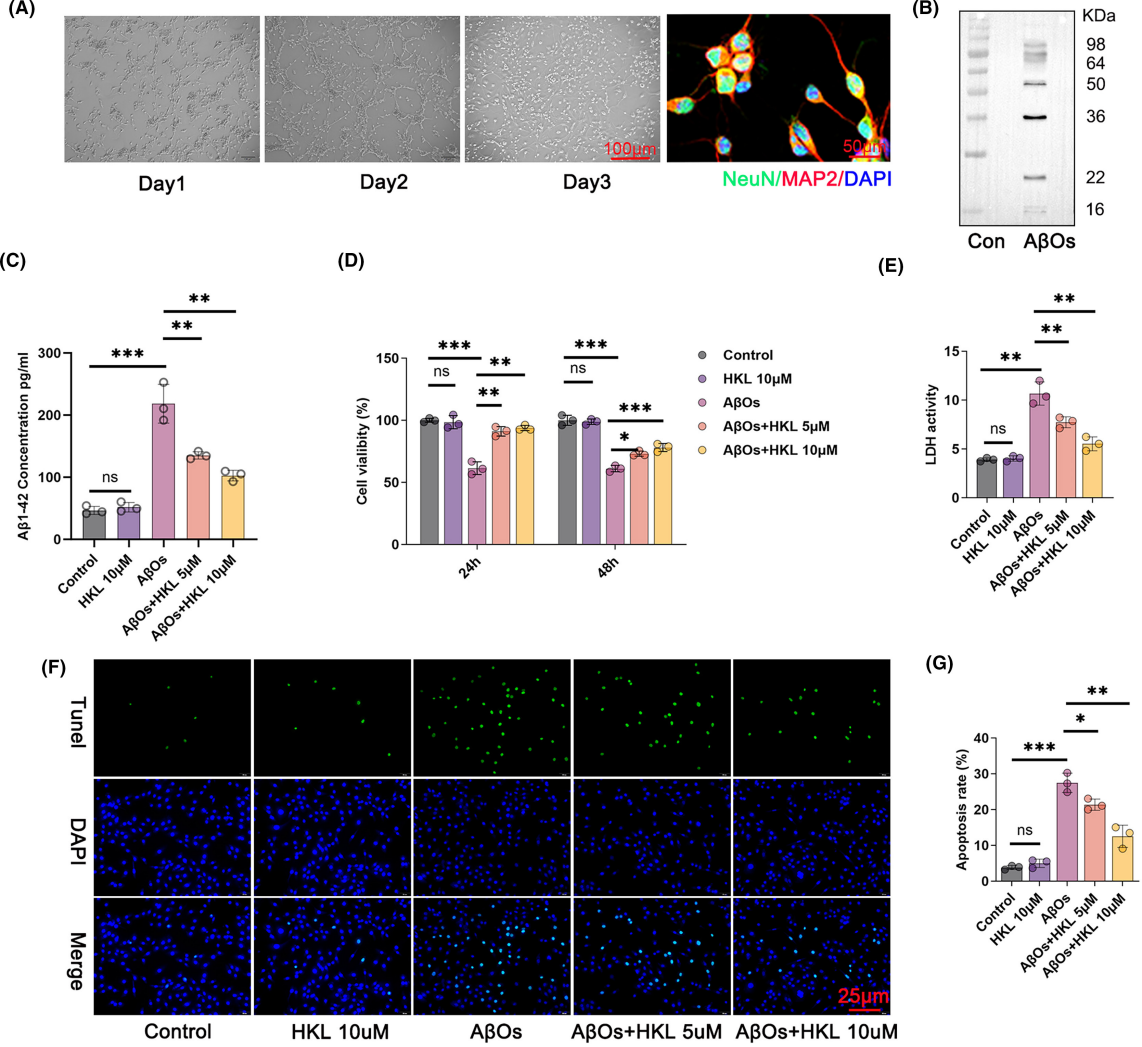
HKL relieved hippocampal neuronal damage and intracellular Aβ aggregation in the hippocampal neuronal model of AD. (A) NeuN/MAP2 staining confirmed the successful isolation of primary mouse hippocampal neurons because they were capable of expressing NeuN and MAP2. (B) Western blot indicated the successful preparation of AβOs. (C) ELISA revealed the inhibitory effect of HKL on intracellular Aβ aggregation in the AβO‐induced hippocampal neuronal model of AD. (D) CCK‐8 assay revealed that HKL treatment improved the viability of the AβO‐induced hippocampal neuronal model of AD. (E) HKL treatment weakened the LDH activity of the AβO‐induced hippocampal neuronal model of AD. (F, G) TUNEL staining suggested the suppressive role of HKL on the apoptosis of the AβO‐induced hippocampal neuronal model of AD. *p < 0.05, **p < 0.01, ***p < 0.001. “ns” represented differences that were not statistically significant.

First, primary mouse hippocampal neurons were isolated. NeuN/MAP2 staining revealed the expression of NeuN and MAP2 in the isolated primary mouse hippocampal neurons (Figure 3A). This phenomenon suggested the successful generation of primary mouse hippocampal neurons.

Next, we prepared AβOs and verified them using Western blot. AβOs mainly comprised 9 monomers and 12 monomers, with a molecular weight of 30–50 kDa (Figure 3B). This suggested the successful preparation of AβOs. They were used to treat primary mouse hippocampal neurons.

Subsequently, we used AβOs (1 μM) to treat primary mouse hippocampal neurons to construct the hippocampal neuronal model of AD. Furthermore, 10 μM HKL was used to treat primary mouse hippocampal neurons to evaluate toxicity. The AβOs (1 μM)‐induced primary mouse hippocampal neurons were treated by HKL at concentrations of 5 and 10 μM, respectively. To evaluate Aβ_1–42_ accumulation in the hippocampal neurons, its concentration in the hippocampal neurons was measured using ELISA. Compared with the control group, the hippocampal neuronal model of AD had considerably higher Aβ_1–42_ concentration (*p* < 0.001). Interestingly, HKL treatment (5 and 10 μM) markedly decreased Aβ_1–42_ concentration in the hippocampal neuronal model of AD (*p* < 0.01) (Figure 3C: *F* = 62.86, df = 4).

Based on CCK‐8 assay, after 24 and 48 h, the primary mouse hippocampal neurons treated with HKL (10 μM) had viability similar to the control group; on the other hand, treatment with AβOs (1 μM) significantly attenuated the viability of primary mouse hippocampal neurons (*p* < 0.001). Therefore, this confirmed the successful establishment of the hippocampal neuronal model of AD, and 10 μM HKL was nontoxic to primary mouse hippocampal neurons. Thereafter, were treated the hippocampal neuronal model of AD with 5 and 10 μM HKL. Both concentration groups markedly increased the viability of the hippocampal neuronal model of AD (*p* < 0.05, *p* < 0.01 or *p* < 0.001) (Figure 3D: *F* = 49.64, df = 4 for 24 h; *F* = 99.62, df = 4 for 48 h). This suggested that HKL could improve the viability of the hippocampal neuronal model of AD.

Next, we measured the LDH activity in the culture medium of the hippocampal neurons in each group using a commercial LDH activity assay kit. The hippocampal neuronal model of AD (induced with 1 μM AβOs) exhibited a distinctly increased LDH activity compared with the control group (*p* < 0.01). On the other hand, HKL (5 and 10 μM) dramatically decreased the LDH activity of the hippocampal neuronal model of AD (*p* < 0.01) (Figure 3E: *F* = 51.82, df = 4).

TUNEL staining was performed to elucidate the apoptosis of hippocampal neurons. Compared with the control group, the hippocampal neuronal model of AD exhibited a considerable increase in the apoptosis rate (*p* < 0.001). However, HKL (5 and 10 μM) significantly decreased AβO (1 μM)‐induced apoptosis (*p* < 0.05 or *p* < 0.01) (Figure 3F,G) (Figure 3G: *F* = 75.35, df = 4). Taken together, these data suggested that HKL could mitigate hippocampal neuronal damage and intracellular Aβ aggregation in the hippocampal neuronal model of AD.

### 3.4 HKL up‐regulated SIRT3 and activated mitochondrial autophagy in the hippocampal neuronal model of AD

**FIGURE 4:**
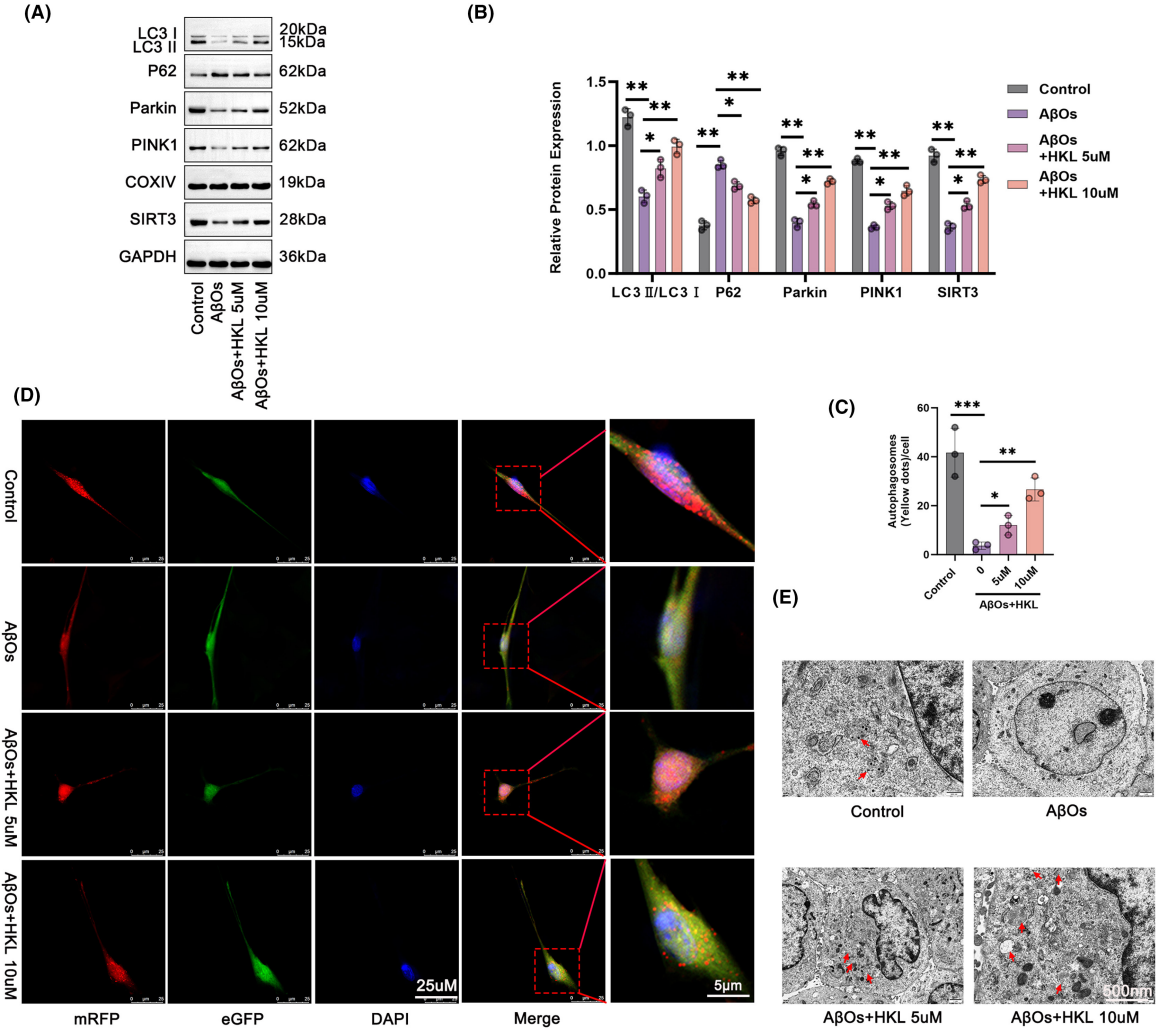
HKL up‐regulated SIRT3 and activated mitochondrial autophagy in the hippocampal neuronal model of AD. (A, B) HKL increased SIRT3, LC3II/LC3I, Parkin, and PINK1 proteins, but decreased P62 protein in the AβO‐induced hippocampal neuronal model of AD. (C, D) mRFP–eGFP–LC3 assay suggested the promoting effect of HKL on the formation of autophagosomes in the AβO‐induced hippocampal neuronal model of AD. (E) TEM revealed that HKL treatment facilitated the formation of autophagosomes in the AβO‐induced hippocampal neuronal model of AD. *p < 0.05, **p < 0.01, ***p < 0.001.

We investigated the effect of HKL on mitochondrial autophagy in the hippocampal neuronal model of AD. First, we evaluated the effect of HKL on SIRT3 expression in the hippocampal neurons because SIRT3 is involved in the regulation of mitochondrial autophagy. Compared with the control group, SIRT3 protein level was considerably decreased in the hippocampal neuronal model of AD (induced by 1 μM AβOs) (*p* < 0.01). In contrast, HKL treatment (5 and 10 μM) remarkably increased SIRT3 expression in the hippocampal neuronal model of AD (*p* < 0.05 or *p* < 0.01). Thereafter, the protein levels of LC3, Parkin, PINK1, and P62 were assessed in the mitochondria of the hippocampal neurons. Compared with the control group, the mitochondria of the hippocampal neuronal model of AD had a lower LC3II/LC3I protein ratio, lower Parkin and PINK1 protein levels, and higher P62 protein levels (*p* < 0.01). However, HKL treatment (5 and 10 μM) reversed this effect of AβOs (1 μM) on the expression of LC3II/LC3I, Parkin, PINK1, and P62 in the mitochondria of the hippocampal neurons (*p* < 0.05 or *p* < 0.01) (Figure 4A,B) (Figure 4B: *F* = 54.63, df = 3 for LC3II/LC3I; *F* = 125.5, df = 3 for P62; *F* = 232.6, df = 3 for Parkin; *F* = 184.4, df = 3 for PINK1; *F* = 130.7, df = 3 for SIRT3).

To observe autophagosomes, we performed the mRFP–eGFP–LC3 assay on hippocampal neurons. Compared with the control group, the hippocampal neuronal model of AD had a much decrease in the number of autophagosomes (*p* < 0.001). Interestingly, this phenomenon was abrogated after HKL treatment (5 and 10 μM) (*p* < 0.05 or *p* < 0.01) (Figure 4C,D) (Figure 4C: *F* = 26.64, df = 3). Similar results were observed in the hippocampal neurons of each group, as demonstrated via TEM (Figure 4E). Therefore, HKL could up‐regulate SIRT3 and activate mitochondrial autophagy in the hippocampal neuronal model of AD.

### 3.5 HKL was conducive to improving the mitochondrial function of the hippocampal neuronal model of AD

**FIGURE 5:**
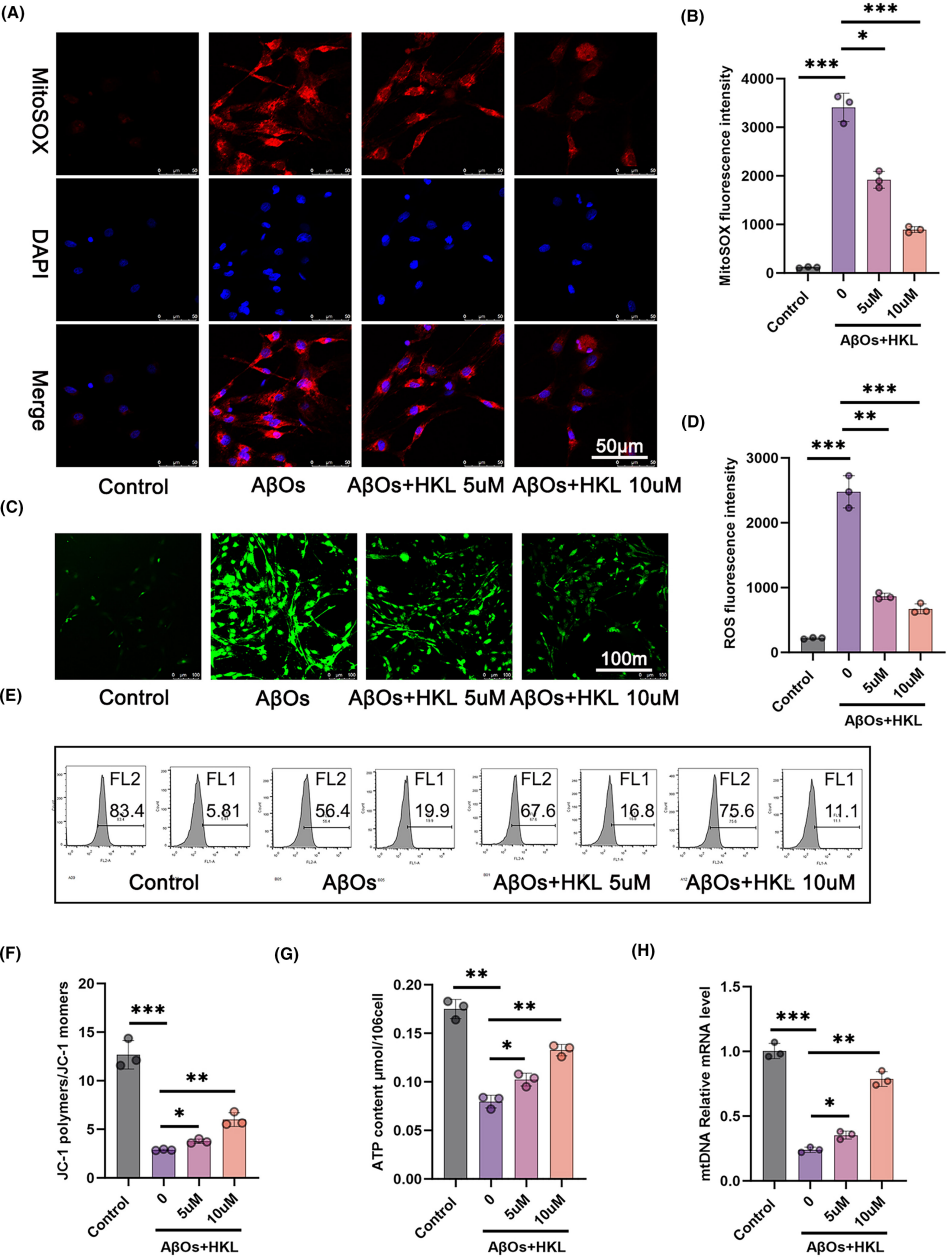
HKL exerted an ameliorative effect on mitochondrial function in the hippocampal neuronal model of AD. (A, B) MitoSOX Red staining revealed the suppressive role of HKL on mitochondrial ROS levels in the AβO‐induced hippocampal neuronal model of AD. (C, D) DCFH‐DA staining revealed that HKL exerted an inhibitory influence on neuronal ROS levels in the AβO‐induced hippocampal neuronal model of AD. (E, F) JC‐1 staining combined with flow cytometry suggested that HKL treatment increased the mitochondrial membrane potential of the AβO‐induced hippocampal neuronal model of AD. (G) HKL treatment enhanced ATP production in the AβO‐induced hippocampal neuronal model of AD. (H) HKL treatment upregulated relative mRNA expression of mtDNA could be upregulated in the AβO‐induced hippocampal neuronal model. *p < 0.05, **p < 0.01, ***p < 0.001.

To explore the effect of HKL on mitochondrial function, we performed MitoSOX Red staining (Figure 5A,B), DCFH‐DA staining (Figure 5C,D), and JC‐1 staining combined with flow cytometry (Figure 5E,F) to measure mitochondrial ROS levels, neuronal ROS levels, and changes in mitochondrial membrane potential, respectively. Compared with the control group, the hippocampal neuronal model of AD (induced with 1 μM AβOs) had significantly increased mitochondrial and mitochondrial ROS levels and decreased mitochondrial membrane potential (*p* < 0.001). Interestingly, HKL treatment (5 and 10 μM) dramatically decreased the mitochondrial and neuronal ROS levels and elevated mitochondrial membrane potential in the hippocampal neuronal model of AD (*p* < 0.05, *p* < 0.01 or *p* < 0.001) (Figure 5B: *F* = 203.5, df = 3; Figure 5D: *F* = 164.6, df = 3; Figure 5F: *F* = 86.63, df = 3).

Additionally, we determined ATP production (Figure 5G) and mtDNA expression (Figure 5H) using a commercial ATP assay kit and qRT‐PCR, respectively. The hippocampal neuronal model of AD had lower ATP content and relative mRNA expression of mtDNA than the control group (*p* < 0.01 or *p* < 0.001). After HKL treatment (5 and 10 μM), the hippocampal neuronal model of AD exhibited a significant increase in ATP content and relative mRNA expression of mtDNA (*p* < 0.05 or *p* < 0.01) (Figure 5G: *F* = 92.74, df = 3; Figure 5H: *F* = 205.2, df = 3). Taken together, the data suggested the ameliorative effect of HKL on the mitochondrial function of the hippocampal neuronal model of AD.

### 3.6 HKL might ameliorate damage to the hippocampal neuronal model of AD by activating mitochondrial autophagy

**FIGURE 6:**
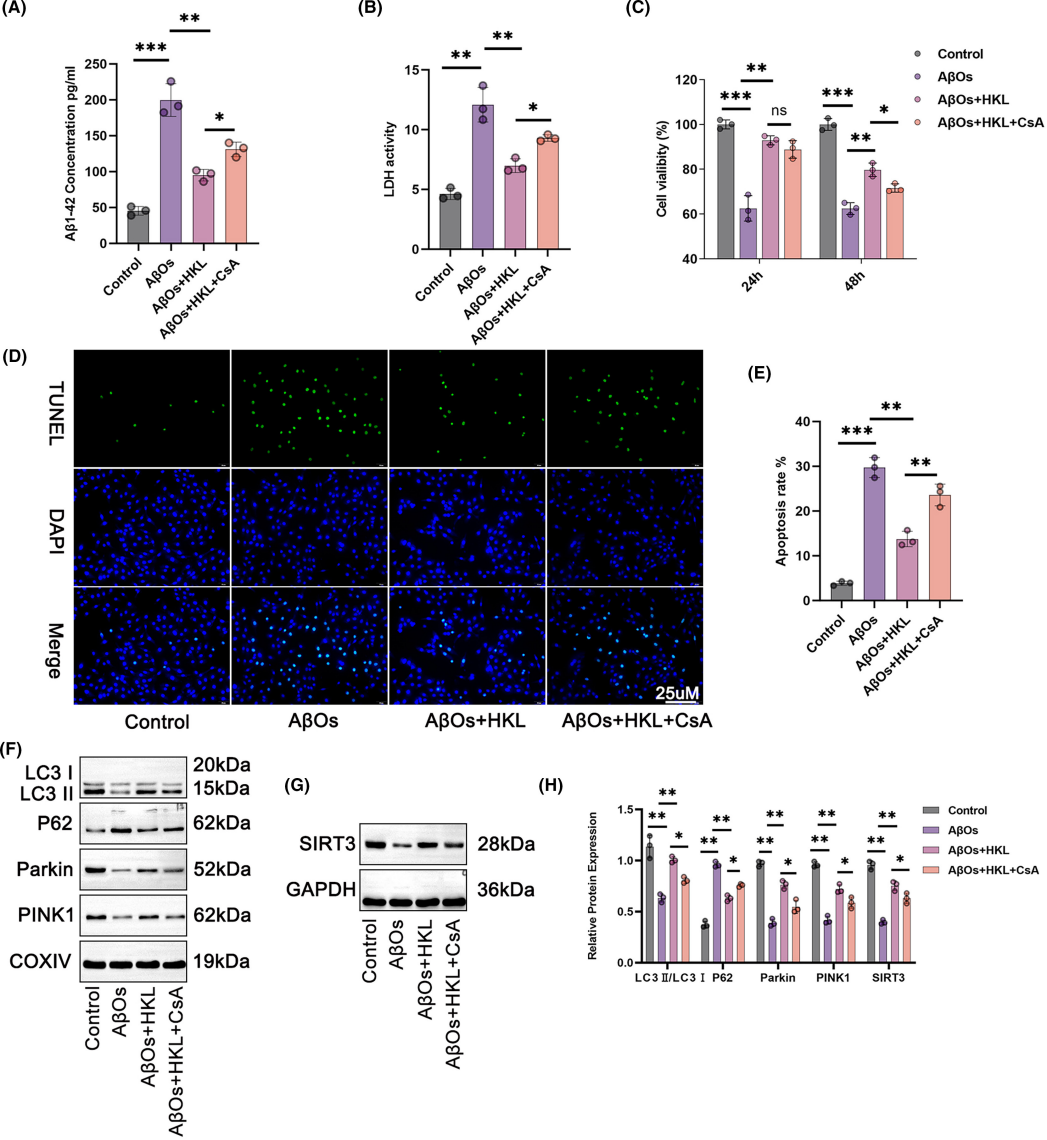
HKL possibly ameliorated damage to the hippocampal neuronal model of AD by activating mitochondrial autophagy. (A) ELISA revealed that CsA treatment abrogated the suppressive role of HKL on intracellular Aβ aggregation in the AβO‐induced hippocampal neuronal model of AD. (B) CsA treatment reversed the inhibition of HKL on LDH activity in the AβO‐induced hippocampal neuronal model of AD. (C) CCK‐8 assay indicated that CsA treatment counteracted the promoting effect of HKL on the viability of the AβO‐induced hippocampal neuronal model. (D, E) TUNEL staining revealed that CsA treatment reversed the repression effect of HKL on the apoptosis of the AβO‐induced hippocampal neuronal model of AD. (F‐H) Western blot suggested that CsA treatment attenuated the activation of SIRT3 and mitochondrial autophagy by HKL in the AβO‐induced hippocampal neuronal model of AD. *p < 0.05, **p < 0.01, ***p < 0.001. “ns” represented differences that were not statistically significant.

Next, we performed rescue experiments by treating the hippocampal neuronal model of AD with 1 μM CsA, a mitochondrial autophagy inhibitor, and 10 μM HKL. As shown in Figure 6A, the intracellular accumulation of Aβ_1–42_ was distinctly exacerbated in the hippocampal neuronal model of AD (induced with 1 μM AβOs, named the AβO group) than in the control group (*p* < 0.001). HKL treatment (the AβO + HKL group) effectively decreased intracellular Aβ_1–42_ accumulation in the hippocampal neuronal model of AD (*p* < 0.01). However, compared with the AβO + HKL group, CsA treatment (the AβO + HKL + CsA group) markedly increased intracellular Aβ_1–42_ concentration in the hippocampal neuronal model of AD (*p* < 0.05) (Figure 6A: *F* = 69.98, df = 3).

Similarly, as shown in Figure 6B, LDH activity was higher in the culture medium of the hippocampal neuronal model of AD than in that of the control group (*p* < 0.01). LDH activity weakened after HKL treatment (*p* < 0.01). However, CsA treatment abrogated the suppressive effect of HKL on LDH activity (*p* < 0.05) (Figure 6B: *F* = 44.78, df = 3).

The CCK‐8 assay revealed the weakened viability of the hippocampal neuronal model of AD compared with the control group (*p* < 0.001). The viability of the hippocampal neuronal model of AD was dramatically increased after HKL treatment (*p* < 0.01); however, this effect of HKL was abrogated by CsA (*p* < 0.05) (Figure 6C) (Figure 6C: *F* = 57.94, df = 3 for 24 h; *F* = 114.9, df = 3 for 48 h).

TUNEL staining revealed that compared with the control group, the apoptosis of the hippocampal neuronal model of AD was exacerbated (*p* < 0.001). HKL treatment considerably decreased the apoptosis of the hippocampal neuronal model of AD (*p* < 0.01); however, this suppressive effect of HKL was counteracted by CsA (*p* < 0.05) (Figure 6D,E) (Figure 6E: *F* = 109.6, df = 3).

To evaluate the degree of mitochondrial autophagy, the levels of autophagy‐related proteins in the mitochondria of hippocampal neurons were assessed using Western blot. Compared with the control group, the hippocampal neuronal model of AD had considerably lower neuronal SIRT3 protein, as well as lower mitochondrial LC3II/LC3I, Parkin, and PINK1 proteins, and higher mitochondrial P62 protein (*p* < 0.01). Undoubtedly, HKL treatment elevated neuronal SIRT3 protein, increased mitochondrial LC3II/LC3I, Parkin, and PINK1 proteins, and decreased mitochondrial P62 protein in the hippocampal neuronal model of AD (*p* < 0.01). However, compared with the AβO + HKL group, the AβO + HKL + CsA group had decreased neuronal SIRT3 protein, reduced mitochondrial LC3II/LC3I, Parkin, and PINK1 protein, and increased mitochondrial P62 protein (*p* < 0.05) (Figure 6F–H) (Figure 6H: *F* = 37.41, df = 3 for LC3II/LC3I; *F* = 252.3, df = 3 for P62; *F* = 99.04, df = 3 for Parkin; *F* = 89.28, df = 3 for PINK1; *F* = 95.44, df = 3 for SIRT3). Therefore, CsA treatment reversed the promoting effect of HKL on damage to the hippocampal neuronal model of AD and the activating effect of HKL on mitochondrial autophagy in the hippocampal neuronal model of AD. Possibly, HKL ameliorated damage to the hippocampal neuronal model of AD by activating mitochondrial autophagy.

### 3.7 HKL might attenuate damage to the hippocampal neuronal model of AD by activating mitochondrial autophagy via up‐regulating SIRT3

**FIGURE 7:**
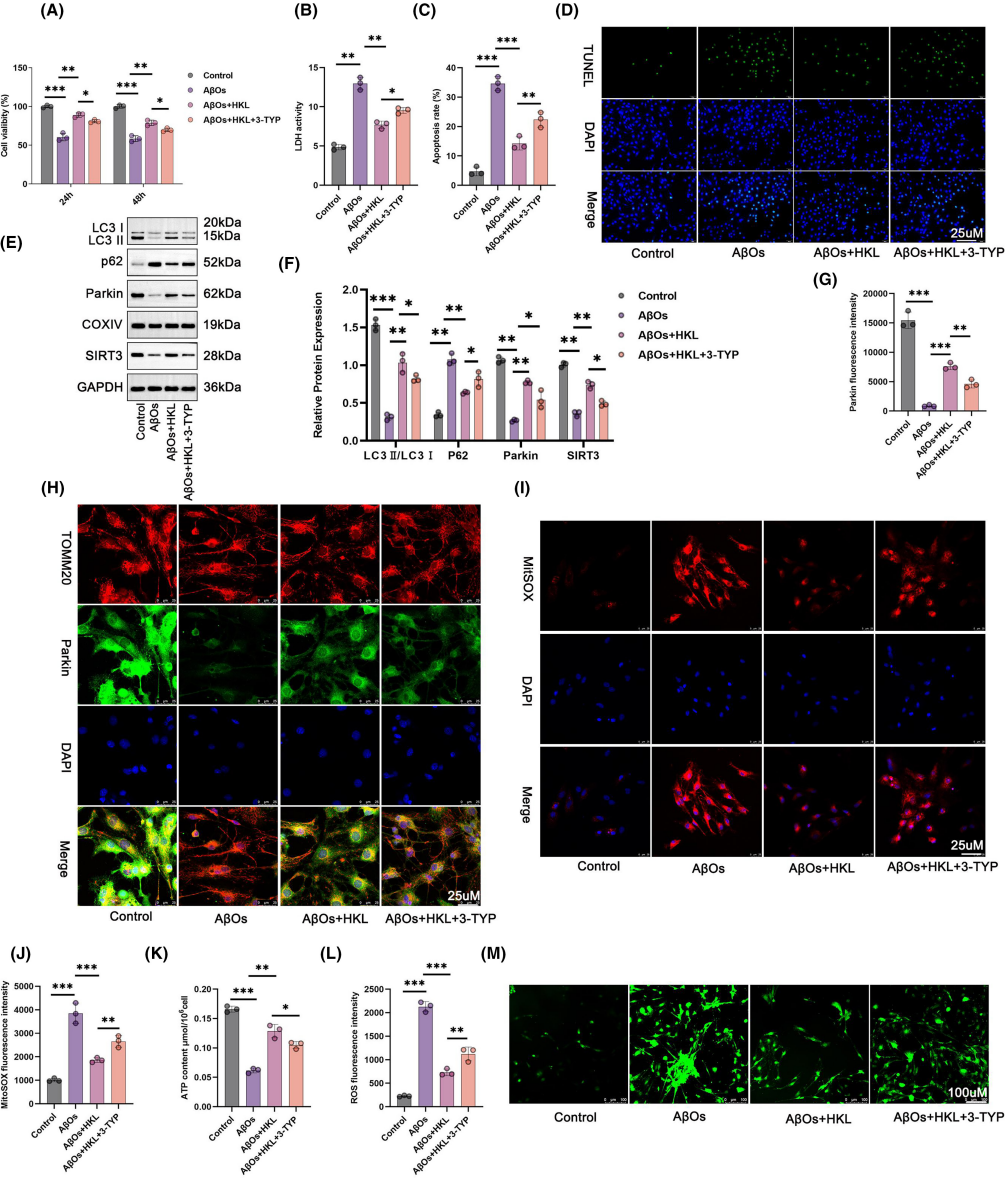
HKL possibly attenuated damage to the hippocampal neuronal model of AD by activating mitochondrial autophagy by upregulating SIRT3. (A) CCK‐8 assay suggested that 3‐TYP treatment reversed the promoting effect of HKL on the viability of the AβO‐induced hippocampal neuronal model of AD. (B) 3‐TYP treatment abrogated the suppression effect of HKL on the LDH activity in the AβO‐induced hippocampal neuronal model of AD. (C, D) TUNEL staining revealed that 3‐TYP treatment counteracted the inhibition of HKL on the apoptosis of the AβO‐induced hippocampal neuronal model of AD. (E, F) Western blot revealed that the activation effect of HKL on SIRT3 expression and mitochondrial autophagy was reversed by 3‐TYP in the AβO‐induced hippocampal neuronal model of AD. (G, H) Immunofluorescence staining revealed that 3‐TYP treatment abrogated the promoting effect of HKL on mitochondrial Parkin expression in the AβO‐induced hippocampal neuronal model of AD. (I, J) MitoSOX Red staining suggested that the suppression effect of HKL on mitochondrial ROS level was abrogated by 3‐TYP in the AβO‐induced hippocampal neuronal model of AD. (K) The enhancement of HKL on ATP production was attenuated by 3‐TYP in the AβO‐induced hippocampal neuronal model of AD. (L, M) DCFH‐DA staining revealed that the repressive role of HKL on neuronal ROS level was reversed by 3‐TYP treatment in the AβO‐induced hippocampal neuronal model of AD. *p < 0.05, **p < 0.01, ***p < 0.001.

The hippocampal neuronal model of AD (induced with 1 μM AβOs) was treated with 20 mM 3‐TYP, a SIRT3 inhibitor, and 10 μM HKL to verify whether HKL relieved damage to the hippocampal neuronal model of AD by regulating SIRT3. The viability of the AβO group was lower than that of the control group (*p* < 0.001). The viability of the hippocampal neuronal model of AD was significantly increased after HKL treatment (the AβO + HKL group) (*p* < 0.01). Interestingly, 3‐TYP treatment (the AβO + HKL + 3‐TYP group) attenuated the viability of the hippocampal neuronal model of AD, when compared with the AβO + HKL group (*p* < 0.05) (Figure 7A: *F* = 85.68, df = 3 for 24 h; *F* = 98.39, df = 3 for 48 h).

Next, we collected the culture medium of the hippocampal neurons in each group to measure LDH activity. Compared with the Control group, the LDH activity of the hippocampal neuronal model of AD was significantly increased (*p* < 0.01). HKL treatment markedly attenuated the LDH activity of the hippocampal neuronal model of AD (*p* < 0.01). However, 3‐TYP treatment reversed this effect of HKL (*p* < 0.05) (Figure 7B: *F* = 123.0, df = 3).

The apoptosis of hippocampal neurons in each group was assessed using TUNEL staining. Compared with the control group, severe apoptosis was observed in the hippocampal neuronal model of AD (*p* < 0.001). After HKL treatment, the apoptosis of the hippocampal neuronal model of AD was significantly mitigated (*p* < 0.001); however, 3‐TYP treatment diminished this suppressive effect of HKL (*p* < 0.01) (Figure 7C,D) (Figure 7C: *F* = 99.30, df = 3).

Western blot revealed lower neuronal SIRT3 and mitochondrial LC3II/LC3I and Parkin protein levels, and higher mitochondrial P62 protein levels in the hippocampal neuronal model of AD when compared with the control group (*p* < 0.01 or *p* < 0.001). The HKL‐treated hippocampal neuronal model of AD exhibited increased neuronal SIRT3 and mitochondrial LC3II/LC3I and Parkin protein levels, but decreased mitochondrial P62 protein levels (*p* < 0.01). Conversely, compared with the AβO + HKL group, the AβO + HKL + 3‐TYP group had lower neuronal SIRT3 and mitochondrial LC3II/LC3I and Parkin protein levels, but higher mitochondrial P62 protein levels (*p* < 0.05) (Figure 7E,F) (Figure 7F: *F* = 121.9, df = 3 for LC3II/LC3I; *F* = 86.45, df = 3 for Parkin; *F* = 67.81, df = 3 for P62; *F* = 174.0, df = 3 for SIRT3).

Besides, compared with the Control group, the fluorescence intensity of mitochondrial Parkin was weakened in the hippocampal neuronal model of AD (*p* < 0.001). HKL treatment remarkably intensified the fluorescence staining of mitochondrial Parkin in the hippocampal neuronal model of AD (*p* < 0.001); however, this enhancing effect of HKL on the fluorescence intensity of mitochondrial Parkin was abrogated by 3‐TYP (*p* < 0.01) (Figure 7G,H) (Figure 7G: *F* = 162.4, df = 3).

We measured mitochondrial ROS levels, ATP production, and neuronal ROS levels via MitoSOX Red staining (Figure 7I,J), a commercial ATP assay kit (Figure 7K), and DCFH‐DA staining (Figure 7L,M), respectively. The hippocampal neuronal model of AD exhibited higher mitochondrial ROS level, lower ATP content, and higher neuronal ROS level than the control group (*p* < 0.001). After HKL treatment, the hippocampal neuronal model of AD had considerably decreased mitochondrial ROS level, increased ATP content, and decreased neuronal ROS level (*p* < 0.01 or *p* < 0.001). However, 3‐TYP treatment counteracted these effects of HKL (*p* < 0.05 or *p* < 0.01) (Figure 7J: *F* = 66.44, df = 3; Figure 7K: *F* = 109.9, df = 3; Figure 7L: *F* = 202.0, df = 3). Taken together, these findings suggested that HKL relieved damage to the hippocampal neuronal model of AD by activating mitochondrial autophagy via up‐regulating SIRT3.

### 3.8 HKL relieved cognitive impairment and Aβ_1–42_ plaque deposition in AD mice by activating hippocampal mitochondrial autophagy via up‐regulating SIRT3

**FIGURE 8:**
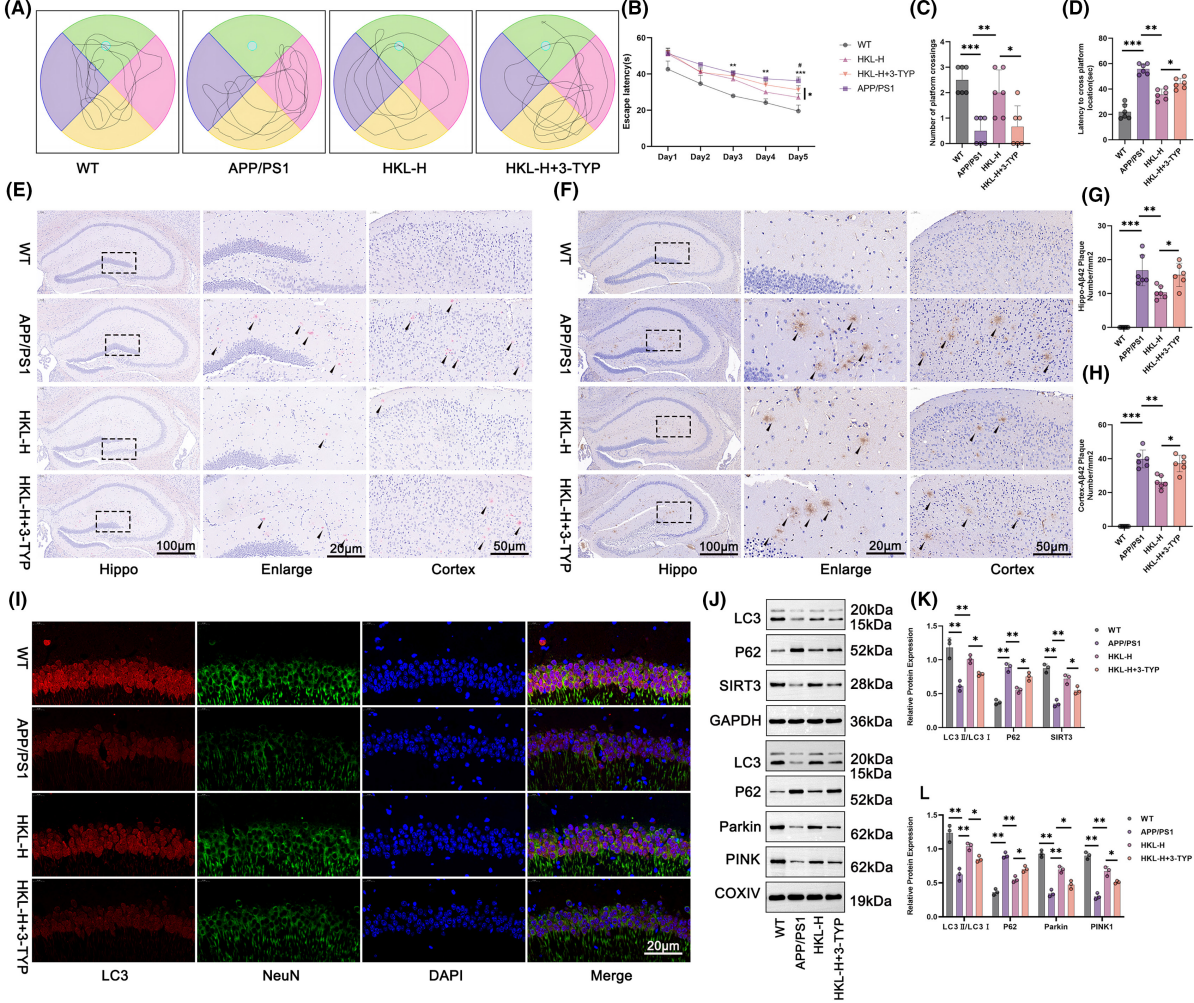
HKL alleviated cognitive impairment and Aβ_1–42_ plaque deposition in AD mice by activating hippocampal mitochondrial autophagy via increasing SIRT3. (A–D) Morris water maze experiment indicated that the alleviation of HKL on cognitive impairment of AD mice was reversed by 3‐TYP. (E) By Congo‐red staining, HKL reduced plaque deposition in hippocampus and cortex of AD mice, whereas 3‐TYP counteracted this effect of HKL. (F–H) Immunohistochemistry revealed that the suppression of HKL on Aβ_1–42_ plaque deposition in hippocampus and cortex of AD mice was abrogated by 3‐TYP. (I) Immunofluorescence staining implied the promotion of HKL on hippocampal autophagy in AD mice, but 3‐TYP eliminated this effect of HKL. (J–L) Base on Western blot, HKL activated hippocampal autophagy and autophagy in hippocampal neuronal mitochondria, whereas this effect was abolished by 3‐TYP. *p < 0.05, **p < 0.01, ***p < 0.001. ^#^ *p* < 0.05 vs. the HKL‐H group.

As for in vivo mechanistic study, this work treated APP/PS1 mice with both high dose of HKL and 3‐TYP (an inhibitor of SIRT3). According to morris water maze experiment, the prolonged escape latency and latency to cross platform location, as well as the reduced number of platform crossing, was observed in APP/PS1 mice, as referring to wild‐type C57BL/6J mice (*p* < 0.01 or *p* < 0.001). High dose of HKL treatment distinctly shortened escape latency and latency to cross platform location, and increased number of platform crossing (the HKL‐H group vs. the APP/PS1 group) (*p* < 0.05 or *p* < 0.01). Conversely, these effects of HKL was eliminated by 3‐TYP, comparatively (the HKL‐H + 3‐TYP group vs. the HKL‐H group) (*p* < 0.05) (Figure 8A–D) (Figure 8B: *F* = 24.45, df = 3; Figure 8C: *F* = 11.29, df = 3; Figure 8D: *F* = 56.37, df = 3).

Congo‐red staining displayed severe plaque deposition in hippocampus and cortex of APP/PS1 mice, whereas high dose of HKL treatment obviously weakened it (the HKL‐H group vs. the APP/PS1 group). Nevertheless, 3‐TYP showed an opposite effect, as it abrogated the mitigating effect of HKL on plaque deposition in hippocampus and cortex of APP/PS1 mice (the HKL‐H + 3‐TYP group vs. the HKL‐H group) (Figure 8E). Based on immunohistochemistry, a large number of Aβ_1–42_ plaques were found in hippocampus and cortex of APP/PS1 mice, as relative to wild‐type C57BL/6J mice (the APP/PS1 group vs. the WT group) (*p* < 0.001). Actually, high dose of HKL treatment resulted in a much decrease in number of Aβ_1–42_ plaques in hippocampus and cortex of APP/PS1 mice (the HKL‐H group vs. the APP/PS1 group) (*p* < 0.01). However, the inhibition of Aβ_1–42_ plaque deposition by high dose of HKL was reversed by 3‐TYP (the HKL‐H + 3‐TYP group vs. the HKL‐H group) (*p* < 0.05) (Figure 8F–H) (Figure 8G: *F* = 39.02, df = 3; Figure 8H: *F* = 119.3, df = 3).

On the exploration of autophagy, immunofluorescence staining displayed the weakened LC3 staining in hippocampus and cortex of APP/PS1 mice, as matched to wild‐type C57BL/6J mice. The intensified LC3 staining in hippocampus and cortex of APP/PS1 mice by high dose of HKL treatment was observed, comparatively (the HKL‐H group vs. the APP/PS1 group). Conversely, this promotion effect of HKL on LC3 expression in hippocampus and cortex of APP/PS1 mice was counteracted by 3‐TYP (Figures 8I and Figure S2).

Additionally, Western blot was for the assessment of autophagy‐related proteins in hippocampus and hippocampal neuronal mitochondria. As a result, APP/PS1 mice expressed lower LC3II/LC3I and SIRT3 proteins and higher P62 protein in hippocampus, as well as lower LC3II/LC3I, Parkin and PINK1 proteins and higher P62 protein in hippocampal neuronal mitochondria, when referring to wild‐type C57BL/6J mice (*p* < 0.01). In contrasted to the APP/PS1 group, APP/PS1 mice in the HKL‐H group possessed higher LC3II/LC3I and SIRT3 proteins and lower P62 protein in hippocampus, as well as higher LC3II/LC3I, Parkin and PINK1 proteins and lower P62 protein in hippocampal neuronal mitochondria (*p* < 0.01). Interestingly, APP/PS1 mice that treated by high dose of HKL and 3‐TYP (the HKL‐H + 3‐TYP group) showed lower LC3II/LC3I and SIRT3 proteins and higher P62 protein in hippocampus, as well as lower LC3II/LC3I, Parkin and PINK1 proteins and higher P62 protein in hippocampal neuronal mitochondria, when compared with those treated by high dose of HKL only (the HKL‐H group) (*p* < 0.05) (Figure 8J–L) (Figure 8K: *F* = 24.58, df = 3 for LC3II/LC3I; *F* = 59.21, df = 3 for P62; *F* = 42.01, df = 3 for SIRT3; Figure 8L: *F* = 29.20, df = 3 for LC3II/LC3I; *F* = 79.80, df = 3 for P62; *F* = 73.96, df = 3 for Parkin; *F* = 82.58, df = 3 for PINK1). Therefore, the activation of HKL on hippocampal mitochondrial autophagy in AD mice was reversed by 3‐TYP. This suggested that HKL might alleviate cognitive impairment and Aβ_1–42_ plaque deposition in AD mice by activating hippocampal mitochondrial autophagy via increasing SIRT3 expression.

## 4 DISCUSSION

In the present study, we treated the mouse model of AD and the hippocampal neuronal model of AD with HKL. HKL was instructed to be effective in relieving AD, possibly by mitigating hippocampal neuronal damage via activating the SIRT3‐mediated mitochondrial autophagy.

Aβ_1–42_ aggregation is a hallmark feature of AD, and the neurotoxicity of soluble oligomers in Aβ_42_ is a major pathogenic factor in AD.^49^, ^50^ This article suggested the improvement of HKL on cognitive performance and the suppression on Aβ_1–42_ aggregation in hippocampus of AD mice. Besides, the repressive role of HKL on Aβ_1–42_ aggregation in the hippocampal neuronal model of AD was also revealed. A study has revealed that HKL possesses the ability to enhance neuronal survival and mitigate the apoptosis of the hippocampal neuronal in rats after traumatic brain injury.^51^ In the AβO‐induced mouse model of AD, HKL administration may relieve the apoptosis of hippocampal neurons in a dose‐dependent manner.^21^ Similarly, in the present study, we suggested that HKL can increase neuronal survival and decrease the apoptosis of the AβO‐induced hippocampal neuronal model of AD. We observed that the LDH activity of the hippocampal neuronal model of AD was increased and that HKL treatment suppressed this increase. LDH can convert pyruvate (the end product of glycolysis) to lactate; however, patients with AD have abnormally elevated lactate levels.^52^ Persistently increased lactate metabolism in neurons can facilitate ROS production, mitochondrial damage, and ultimately neuronal damage.^53^ Previous studies have reported the inhibitory effect of HKL on LDH activity in diseases such as myocardial dysfunction and myocardial ischemia–reperfusion injury.^54^, ^55^ HKL can be a potential agent for treating neurodegenerative diseases because it suppresses LDH release and alleviates mitochondrial dysfunction and excitotoxicity in neurons.^56^, ^57^ Therefore, our findings suggest that HKL relieves hippocampal neuronal damage in AD by suppressing LDH activity and Aβ_1–42_ aggregation.

Mitochondrial dysfunction plays a central role in AD.^58^ Mitochondrial autophagy homeostasis is needed for maintaining normal mitochondrial function, cell survival, and normal cellular and biological functions.^59^ In the present study, we observed that mitochondrial autophagy was suppressed in the mouse model of AD and the hippocampal neuronal model of AD. Interestingly, HKL treatment increased the levels of autophagy‐promoting proteins (including LC3II/LC3I, Parkin, and PINK1) but decreased that of the autophagy‐inhibition protein P62 in the hippocampal mitochondria of the mouse model of AD and the hippocampal neuronal model of AD. Furthermore, HKL treatment facilitated autophagosome formation in the hippocampal neuronal model of AD. Neuronal autophagy is important under both normal physiological and pathological conditions, and dysregulated autophagy facilitates AD progression.^60^ Autophagy stimulation in AD can be a promising strategy for treating AD.^58^ In the present study, HKL treatment activated the suppressed mitochondrial autophagy in neurons of the AD mouse model and the hippocampal neuronal AD model. HKL can improve cognitive impairment in mice by relieving mitochondrial dysfunction and activating mitophagy.^22^ Mitophagy is a vital form of autophagy and is crucial for eliminating mitochondrial dysfunction.^61^ We observed that HKL activated mitochondrial autophagy in the AD mouse model and the hippocampal neuronal AD model. Mitochondrial dysfunction can induce ROS production, ultimately resulting in neuronal death.^62^ HKL can decrease mitochondrial ROS production in hippocampal neurons to decrease cognitive impairment in mice.^63^ In the present study, we suggested that HKL exerts an inhibitory role on mitochondrial and neuronal ROS production in the AD mouse model and the hippocampal neuronal AD model. Simultaneously, HKL was conducive to restoring mitochondrial function in AD because it elevated mitochondrial membrane potential, ATP production, and mtDNA expression in the hippocampal neuronal model of AD. A previous study has reported that HKL treatment can mitigate mitochondrial damage and increase the expression of autophagy markers such as LC3‐II, Parkin, and PINK1 in the hippocampus to relieve cognitive impairment in mice; however, treatment with 3‐MA, an autophagy inhibitor, abrogated these neuroprotective effects of HKL.^63^ Similarly, in the present study, CsA, a mitochondrial autophagy inhibitor, reversed the promoting effects of HKL on mitochondrial autophagy and the protective effect on the hippocampal neuronal model of AD. Therefore, these findings suggest that HKL exerts a neuroprotective effect on AD by activating mitochondrial autophagy. To the best of our knowledge, this was the first study to elucidate the effect of HKL on mitochondrial autophagy in AD.

HKL is an agonist of SIRT3.^64^ We observed that SIRT3 protein levels were decreased in the AD mouse model and the hippocampal neuronal AD model. Interestingly, HKL elevated SIRT3 protein levels in both of the animal and cell models of AD. Accumulating evidence suggests that SIRT3 dysfunction is closely associated with AD pathologies, and SIRT3 activation can be a promising strategy for ameliorating AD.^65^ SIRT3 is conducive to relieving the apoptosis of ischemic neurons by enhancing mitochondrial autophagy.^66^ In the present study, treatment with 3‐TYP, a SIRT3 inhibitor, abrogated the protective effect of HKL on the AD mouse model and the hippocampal neuronal AD model. Furthermore, it counteracted the activation effect of HKL on mitochondrial autophagy in the AD mouse model and the hippocampal neuronal AD model. Therefore, HKL may relieve damage to the hippocampal neurons in AD by activating mitochondrial autophagy by upregulating SIRT3 expression. A previous study has reported that HKL can attenuate cognitive deficits in mice with AD by activating mitochondrial SIRT3.^25^ Moreover, HKL can attenuate the effect of Aβ on AD by increasing SIRT3 expression, decreasing ROS production, and improving mitochondrial function; therefore, it can be an effective compound for AD treatment.^67^ Meanwhile, HKL can ameliorate mitochondrial dysfunction and neuronal damage, and activate mitophagy in AβO‐induced hippocampal neurons in a SIRT3‐dependent manner.^22^ Our study findings are similar to those of these studies. To the best of our knowledge, this is the first study that suggested that HKL can mitigate hippocampal neuronal damage in AD by activating the SIRT3‐mediated mitochondrial autophagy.

## 5 CONCLUSION

In this work, APP/PS1 mice were used as animal AD models and treated with HKL. HKL improved cognitive performance of AD mice; mitigated hippocampal Aβ_1–42_ deposition; and promoted hippocampal neuron survival in AD mice. By in vitro experiments, the hippocampal neuronal model of AD was established and treated by HKL. HKL could relieve damage to the hippocampal neuronal model of AD. Mechanically, HKL might relieve AD by protecting hippocampal neurons from damage via activating the SIRT3‐mediated mitochondrial autophagy. Therefore, HKL may be an effective agent for treating AD in clinical settings.

## AUTHOR CONTRIBUTIONS

Haitao Li conceived and designed the research; Yishu Yang, Jinmei Sun, Wei Zhang, Yili Wu and Yuanruhua Tian analyzed and interpreted the data. All authors were involved in drafting and revising the manuscript.

## FUNDING INFORMATION

Funding from Key Laboratory of Alzheimer's Disease of Zhejiang Province, Institute of Aging, Wenzhou Medical University (No. ZJAD‐2021003). Ten major professional constructions of Beijing Medical Management Center (No. Q19051‐07) Hospital/Ten Majors/Special Financial Fund.

## CONFLICT OF INTEREST STATEMENT

The authors have no conflict of interest to report.

## Supporting information


Figure S1

Figure S2

## Data Availability

The data that support the findings of this study are available in the methods and/or supplementary material of this article. The data that support the findings of this study are available from the corresponding author upon reasonable request.
