# Supplementary figures and images for: Honokiol relieves hippocampal neuronal damage in Alzheimer's disease by activating the SIRT3‐mediated mitochondrial autophagy
Source: CNS Neurosci Ther. 2024 Aug 4;30(8):e14878. doi: 10.1111/cns.14878 (PMC11298204; doi:10.1111/cns.14878)

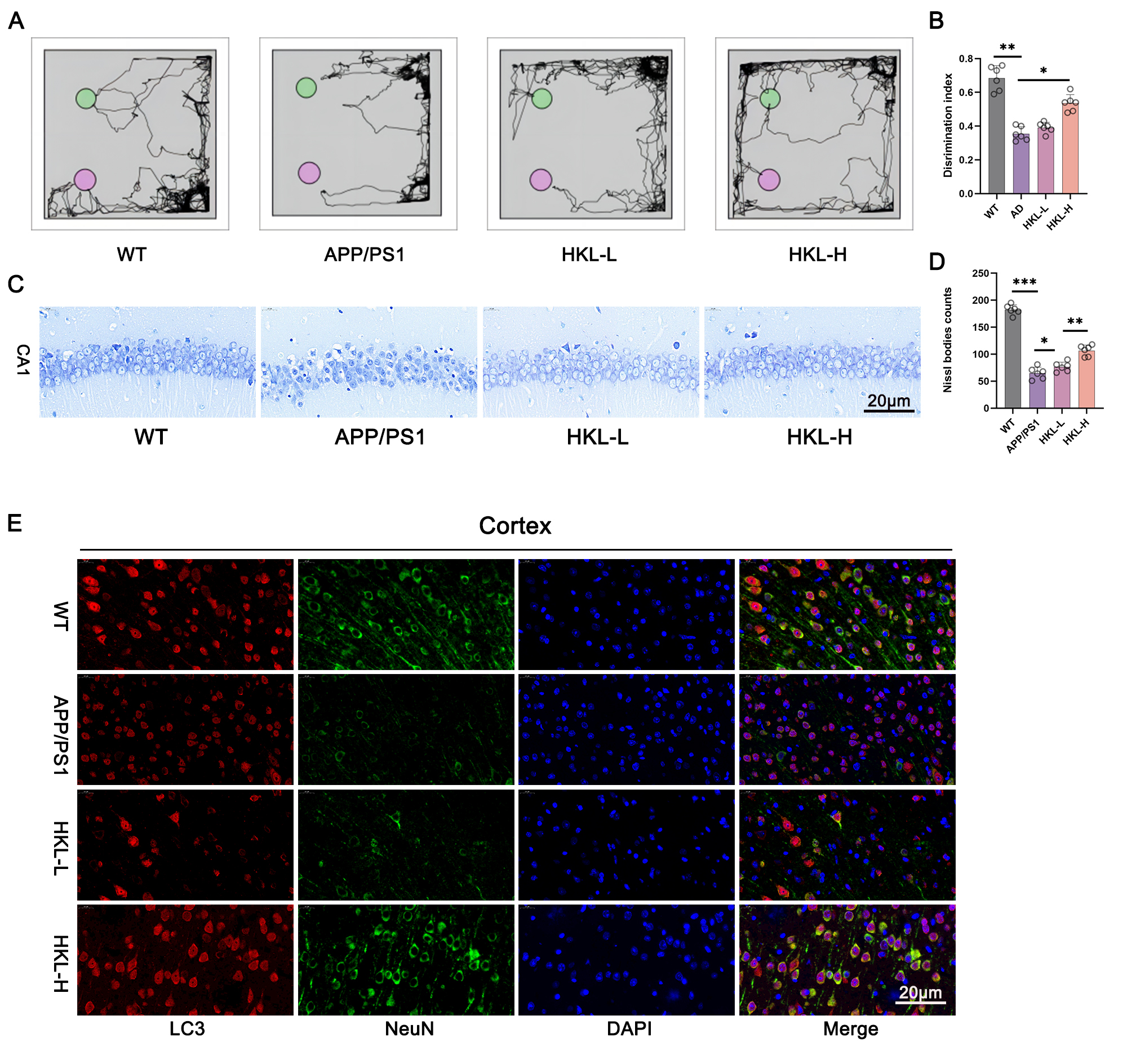

Supplement: Supplementary file 1 — Figure S1 [file CNS-30-e14878-s002.jpg]

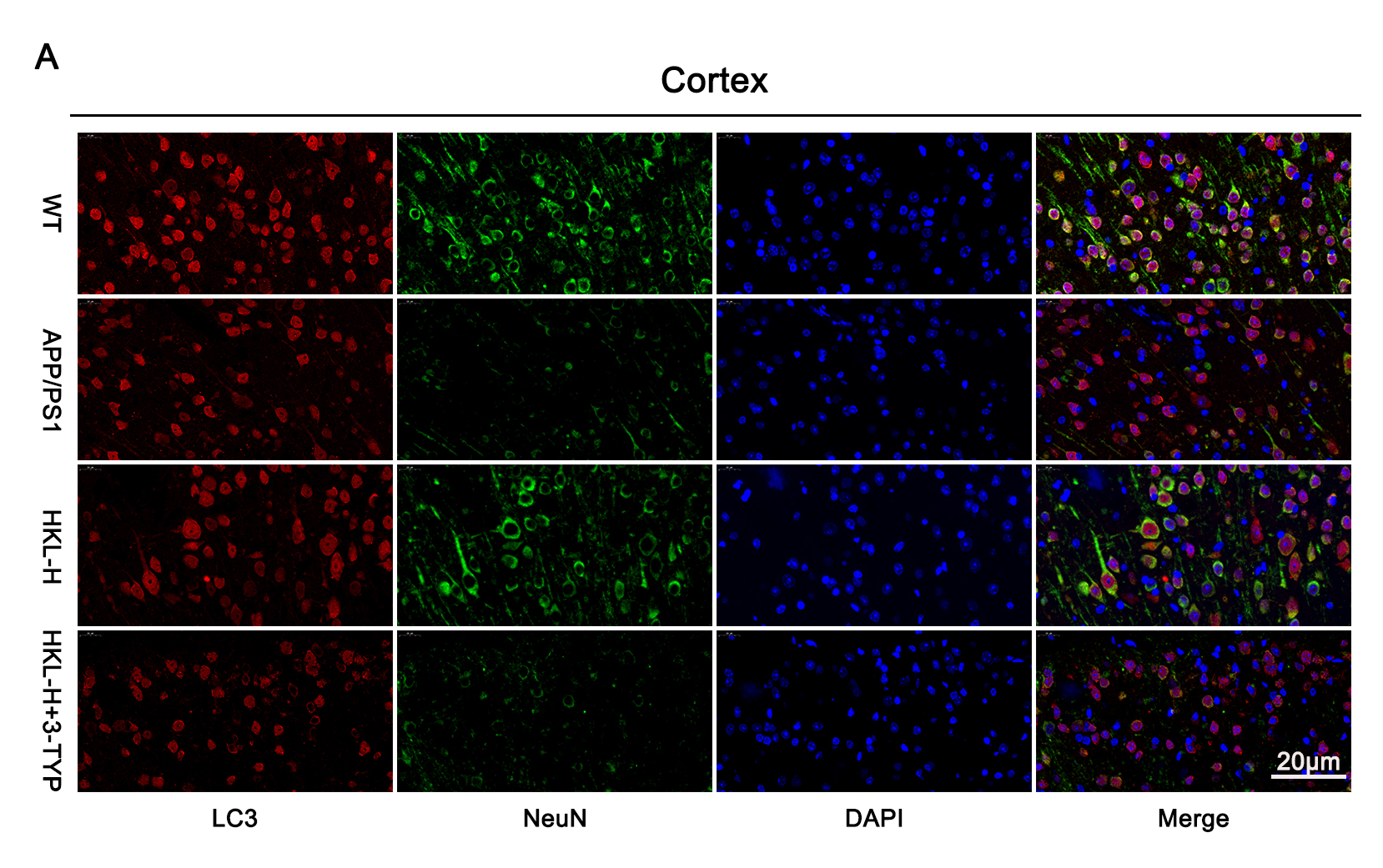

Supplement: Supplementary file 2 — Figure S2 [file CNS-30-e14878-s001.jpg]
